# Effects of Long-Term Triclosan Exposure on Microbiota in Zebrafish

**DOI:** 10.3389/fmicb.2021.604313

**Published:** 2021-10-12

**Authors:** Ning Tang, Pianpian Fan, Xiaogang Yu, Rui Ma, Yexuan Tao, Weiye Wang, Fengxiu Ouyang

**Affiliations:** ^1^Ministry of Education and Shanghai Key Laboratory of Children’s Environmental Health, Xinhua Hospital, Shanghai Jiao Tong University School of Medicine, Shanghai, China; ^2^Department of Clinical Nutrition, Xinhua Hospital, Shanghai Jiao Tong University School of Medicine, Shanghai, China

**Keywords:** Triclosan (TCS), long-term exposure, microbiota in the gastrointestinal tract, 16S rRNA gene, early life, zebrafish

## Abstract

**Background:** Triclosan (TCS) is a widely used antibacterial agent in personal care products and is ubiquitous in the environment. We aimed to examine whether TCS exposure affects microbiota in the gastrointestinal tract of zebrafish.

**Methods:** After exposure to TCS 0 (Dimethyl Sulphoxide, DMSO control), 0.03, 0.3, 3, 30, 100, and 300ng/ml, respectively, from day 0 to 120days post fertilization (dpf), or for 7days in adult 4-month zebrafish, the long- and short-term impact of TCS exposure on the microbiome in the gastrointestinal tract was evaluated by analyzing 16S rRNA gene V3-V4 region sequencing.

**Results:** The top two most dominant microbiota phyla were *Proteobacteria* and *Fusobacteria* phylum in all zebrafish groups. In TCS exposure 0–120 dpf, compared with DMSO control, the mean number of microbial operational taxonomic units (OTUs) was 54.46 lower (*p*<0.0001), Chao indice 41.40 lower (*p*=0.0004), and Ace indice 34.10 lower (*p*=0.0044) in TCS 300ng/ml group, but no change was observed in most of the other TCS concentrations. PCoA diagram showed that the microbial community in the long-term TCS 300ng/ml exposure group clustered differently from those in the DMSO control and other TCS exposure groups. A shorter body length of the zebrafish was observed in the long-term TCS exposure at 0.03, 100, and 300ng/ml. For 7-day short-term exposure in adult zebrafish, no difference was observed in alpha or beta diversity of microbiota nor the relative abundance of *Proteobacteria* or *Fusobacteria* phylum among DMSO control and any TCS levels, but a minor difference in microbial composition was observed for TCS exposure.

**Conclusions:** Long-term exposure to high TCS concentration in a window from early embryonic life to early adulthood may reduce diversity and alter the composition of microbiota in the gastrointestinal tract. The effect of short-term TCS exposure was not observed on the diversity of microbiota but there was a minor change of microbial composition in adult zebrafish with TCS exposure.

## Introduction

Triclosan (TCS) is a widely used antimicrobial compound in over 2000 consumer products (Halden, 2014). It inhibits or destroys a wide range of bacteria *via* the inhibition of bacterial fatty acid biosynthesis (Yee and Gilbert, 2016). TCS is mainly discharged into sewage after usage of daily products, and can be detected in most seawater, river, and even drinking water all over the world, ranging from 0.03ng/l to 1,023ng/l (Peng et al., 2008; Kumar et al., 2010; Li et al., 2010). This may create environmental pollution and public health hazards. TCS enters the human body mainly by oral intake/ gastrointestinal absorption, and skin contact (Dann and Hontela, 2011). TCS has been detected in most human bio-samples including urine, blood, breast milk, and cord blood samples (Calafat et al., 2008; Halden, 2014; Wang et al., 2017). The median TCS concentration was about 3ng/ml in urine samples in China (Wang et al., 2017). Despite its antimicrobial activity and being ubiquitous in the aquatic environment, it is unclear whether TCS exposure affects the diversity and composition of the gut microbiome in human and aquatic fish. Few studies have examined the effect of TCS on gut microbiota, and research is especially scarce on long-term exposure from early life (Halden, 2016).

Early life is a period critical for the initiation and development of gut microbiota, which is known to be related to a variety of health outcomes (Wen and Duffy, 2017; Robertson et al., 2019). Previous studies have found that high TCS exposure may decrease the body length in zebrafish embryos (Kim et al., 2018), and alter the diversity of microbiota in a short exposure time (Gaulke et al., 2016; Oliveira et al., 2016). In human studies, the alpha-diversity of the fecal microbiome was reduced in infants fed with breast milk of detectable TCS (Bever et al., 2018). In addition, TCS was also found to induce antibiotic-resistant species in the gut microbiome of mothers and infants who used TCS-containing toothpaste daily (Ribado et al., 2017). In animal studies, TCS exposure from birth to 9weeks might change the relative abundance of gut microbiota at the family level in rats (Hu et al., 2016). Another study found that exposure to TCS for 13weeks decreased the alpha-diversity and composition of the gut microbiome in young mice (Gao et al., 2017). To date, few studies have examined TCS exposure on the gut microbiome in fish, and two studies have focused mainly on the short-term (one is diet exposure to 100μg/g TCS for four and seven days on adult zebrafish, and the other is exposed to TCS 100ng/l and 1,000ng/l solution for seven days on fathead minnow larvae; Narrowe et al., 2015; Gaulke et al., 2016). Both studies showed that short-term exposure to TCS might alter the gut microbiome (Narrowe et al., 2015; Gaulke et al., 2016).

Zebrafish is a well-established aquatic animal model with well-known ecological dynamics of gut microbial communities (Jemielita et al., 2014; Stephens et al., 2015, 2016). In zebrafish, gut microbiota become similar to the adult period from 75days post-hatching (dph; Stephens et al., 2016), and zebrafish reach sexual maturity at about 120days post fertilization (dpf; Balasubramani and Pandian, 2008). In this study, we aimed to examine the effect of long-term (starting from early life) exposure to TCS on the microbiota in the gastrointestinal tract of zebrafish. We also examined the effect of short-term TCS exposure on microbiota in adult zebrafish that grew without TCS.

## Materials and Methods

### Triclosan

Triclosan (Irgasan, 5-chloro-2-(2,4-dichlorophenoxy), purity ≥97.0%) and Dimethyl Sulfoxide (DMSO, purity ≥99.9%) were purchased from Sigma-Aldrich (St. Louis, MO, United States).

### Zebrafish Strains, the Process for Obtaining Fertilized Eggs and Zebrafish Maintenance

The zebrafish wild type strain AB (*Danio rerio*) were used in this study. Male and female zebrafish aged 5-months-old were acclimated in glass tanks containing regular fish-raising water (without TCS) under a photoperiod of 14:10h light/dark cycle for 4weeks. Zebrafish were fed with brine shrimp twice per day at 09:00 and 18:00h. At 30min after feeding, the remaining food and feces were removed.

The fertilized eggs of zebrafish were collected within 30min after natural mating, rinsed with the regular fish-raising water (without triclosan). Unfertilized eggs were discarded. Zebrafish larvae were fed with paramecium twice per day from 3 dpf till 14 dpf. After that, zebrafish larvae were fed with brine shrimp from 15 dpf. The pH was maintained at 7.5±0.5, temperature maintained at 28±0.5°C, conductivity maintained at 550±50 μS, and dissolved oxygen of the exposure media were monitored.

### TCS Exposure Levels

The exposure doses were set at 9 levels of TCS exposure from 0 (DMSO as solvent control, and regular fish-raising water as blank control), 0.03, 0.3, 3, 30, 100, 300 to 900ng/ml, with consideration of the exposure levels of human, fish and the aquatic environment (Oliveira et al., 2009; Wang et al., 2017; Nag et al., 2018). Concentrations of 0.03–3ng/ml represented TCS levels in rivers, lakes, and wastewater (Kumar et al., 2010; Nag et al., 2018), the concentration of 3–100ng/ml represented median and highest TCS levels in urinary samples from human populations (Philippat et al., 2012; Wang et al., 2017). DMSO was used as a solvent to enhance TCS solubility because TCS has low water solubility (Goldsmith, 2004), and the concentration of DMSO in both DMSO control and the TCS experimental groups was set to 0.01%. Zebrafish embryos are tolerant to 0.01% concentrations of DMSO (Lantz-McPeak et al., 2015).

### Long-Term Exposure to TCS Starting From Early Life

To examine the effect of long-term exposure from early life, TCS exposure was started in fertilized zebrafish eggs. Fertilized zebrafish eggs were collected and cultured at 28.0±0.5°C under 14:10 light/dark photoperiod cycle in a climate chamber. We collected fertilized eggs within 30min post fertilization and randomly assigned 270 fertilized zebrafish eggs to the 9 TCS exposure groups (30 eggs per TCS exposure group) and raised to 120 dpf. Specifically, we put 30 fertilized zebrafish eggs randomly in each culture dish with 100ml medium. At 14 dpf, zebrafish were transferred from dishes into tanks and continued to culture with 1,000ml exposure medium up to 120 dpf. We changed half of the medium each time twice per day. All glass containers were autoclaved prior to use and all fish tanks were placed in the same room to assume similar environmental conditions.

After exposure to TCS/or control, respectively, for 120 dpf, the fish were euthanized by ice-cold water bath immersion on the 120th dpf morning before feeding. The surface of each fish was sterilized using 75% ethanol and then the gastrointestinal tract (from esophagus to anus; Gaulke et al., 2016) with feces gently removed with a sterile tweezer and surgical blade, and put into a sterile DNAse free tube and stored at −80°C until processing.

### Short-term Exposure to TCS in Adult Zebrafish

In addition, to examine the change in microbiota in the gastrointestinal tract of zebrafish under short-term exposure, we exposed adult zebrafish (4months of age) to the same 9 TCS exposures levels (12 fish per group, 6 male and 6 female) as long-term groups for 7days. The gastrointestinal tract samples were also collected with the same sterile method as the long-term exposure groups.

### Physical Measurement

In the long-term exposure study, we measured and recorded the body weight and length of zebrafish at 120 dpf. After the zebrafish were euthanized by ice water, dry weight and the length from the anterior mouth to the tail tip were measured.

### DNA Extraction, 16S rRNA Gene Sequencing

Microbial DNA was extracted from the gastrointestinal tract samples of zebrafish using the beads-beating method as previously described (Godon et al., 1997). A detailed description of the methods is available in the Supplementary Material (Appendix 1). The V3–V4 region of the bacterial 16S ribosomal RNA (16S rRNA) gene was amplified by PCR using bar-coded universal primers F1 and R2 (5′- CCTACGGGNGGCWGCAG-3′ and 5′-GACTACHVGGGTATCTAATCC-3′) corresponding to positions 341 to 805. In the above primers, “W” represents degenerate bases of A or T, “H” represents bases of A, T or C, and the “V” represents G, A, or C. PCR reactions were carried out with the following recipe: 2μl dNTPs (2.5mm), 5μl 5×FastPfu Buffer, 0.5μl FastPfu Polymerase, 1μl each forward and reverse primer (5μm), 10ng template DNA and PCR-grade water in a final volume of 20μl. Reactions were performed on a GeneAmp® PCR System 9,700 (Thermo Fisher Scientific Inc., Waltham, MA, United States) with conditions: 94°C for 3min followed 94°C for 30s, 55°C for 30s, and 72°C for 30s for 21cycles, with a final extension of 8min at 72°C (Zhang et al., 2016). Amplicons were sequenced using the Illumina Miseq platform (Illumina, San Diego, CA;^1^ miseq.html) with paired-end 300cycle MiSeq Reagent Kit V2 (Illumina; Caporaso et al., 2012).

Throughout the experimental process (i.e., DNA extraction, 16S rRNA gene amplification, and library preparation), nuclease-free materials were used and gloves were changed between handling each sample to prevent accidental cross-contamination between samples. The background negative control was set and subjected to the same DNA extraction procedure and analysis protocol alongside the intestine samples of zebrafish. The lab technicians were blinded to the exposure status of the zebrafish.

### 16S rRNA Gene V3-V4 Sequence Data Analysis

High-quality sequence alignments, sequence clustering, and operational taxonomic unit (OTU) delineation were performed as previously described (Zhang et al., 2019). The Quantitative Insights Into Microbial Ecology (QIIME) platform was used for sequence data analysis. The analysis data were extracted from the raw data using USEARCH 8.0 (Edgar, 2010), and the details of data clean process and filtering criteria are shown in the Supplementary Material (Appendix 2). OTUs were classified based on 97% similarity after the chimeric sequences were removed using UPARSE (version 7.1).^2^ The phylogenetic affiliation of each 16S rRNA gene sequence was analyzed by RDP Classifier^3^ against the Silva (SSU123) 16S rRNA gene database using the confidence threshold of 70% (Amato et al., 2013).

The relative abundances of bacteria were calculated at the levels from phylum to genus. Alpha-diversity indices were calculated and compared between the DMSO control group and exposure groups using linear mixed models. Beta-diversity was evaluated by Principal Coordinate Analysis (PCoA) based on bray-curtis (Gaulke et al., 2016). Sample clustering in beta-diversity analysis was tested by Adonis using the vegan R package.^4^ The linear discriminant analysis (LDA) effect size (LEfSe) analysis was performed to identify features that discriminated among TCS exposure groups. All the statistical analyses on microbiome data were carried out in R (version 3.4.0).

To account for observation correlation within the same TCS exposure tank, linear mixed models were used to evaluate the associations of TCS exposure levels with the diversity and relative abundance of microbiota at phylum and genus levels, and with body weight and length of zebrafish. Statistical analysis was carried out in SAS 9.3 (SAS Institute, Inc., Cary, NC, United States). Two-sided values of *p*<0. 05 were considered statistically significant.

## Result

The number and proportion of male was 4 (50%) in the DMSO control, 2 (28.6%) in the blank control, and 3 (50%), 3 (42.9%), 5 (71.4%), 5 (62.5%), 4 (50%), 3 (42.9%) in TCS 0.03, 0.3, 3, 30, 100, 300ng/ml groups, respectively. There was no statistical difference in male proportion (or sex ratio) among the DMSO control group and other TCS groups. As shown by Kaplan–Meier survival curves (Supplementary Figure S1), overall, there was no difference in mortality among all TCS groups across 0–120 dpf (Log-rank test, *p*=0.988), However, the incidence density of larvae death was higher with higher TCS exposure levels in the first 5days (Log-rank test, *p*=0.012).

### The Effect of Long-Term TCS Exposure on the Alpha-Diversity of Microbiota in the Gastrointestinal Tract

**Figure 1:**
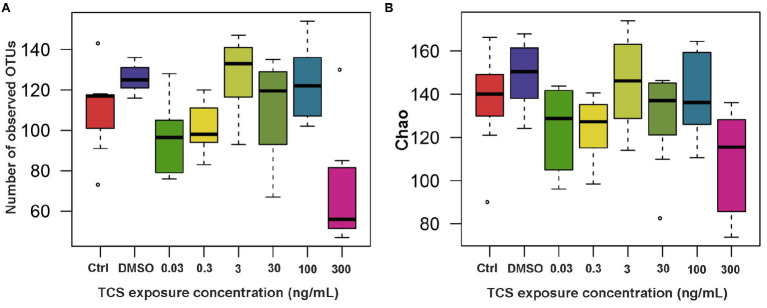
The alpha-diversity (**A**: number of observed OTUs; **B**: Chao index) of microbiota in zebrafish with different concentrations of TCS exposure compared to the control group (DMSO group) at the OTU level. OTUs, Operational Taxonomic Units.

All of the zebrafish at the highest (900ng/ml) TCS group died before 5 dpf, so we did not measure their microbiota. After 120days of TCS exposure, mean (± SE) number of OTUs were 110.00±8.43 in the blank control, 125.75±2.39 in DMSO control, and 96.83±7.91, 101.57±5.13, 126.86±7.43, 110.63±8.45, 123.25±6.49, and 71.29±11.22 in TCS 0.03ng/ml, 0.3ng/ml, 3ng/ml, 30ng/ml, 100ng/ml and 300ng/ml exposure groups, respectively. Linear mixed-model analyses revealed that the mean number of OTUs was 54.46 lower (*p*<0.0001), Chao indice 41.40 lower (mean±SE: 107.62±9.77 vs. 149.02±5.61, *p*=0.0004), and Ace indice 34.10 lower (mean±SE: 112.98±7.50 vs. 147.08±4.44, *p*=0.0044) in TCS 300ng/ml group than the DMSO control group (Figure 1). The Shannon indice (*p*=0.40) and Simpson indice (*p*=0.40) in TCS 300ng/ml group were not significantly different from those in DMSO control group.

### The Effect of Long-Term TCS Exposure on the Beta-Diversity of Microbiota in the Gastrointestinal Tract

**Figure 2:**
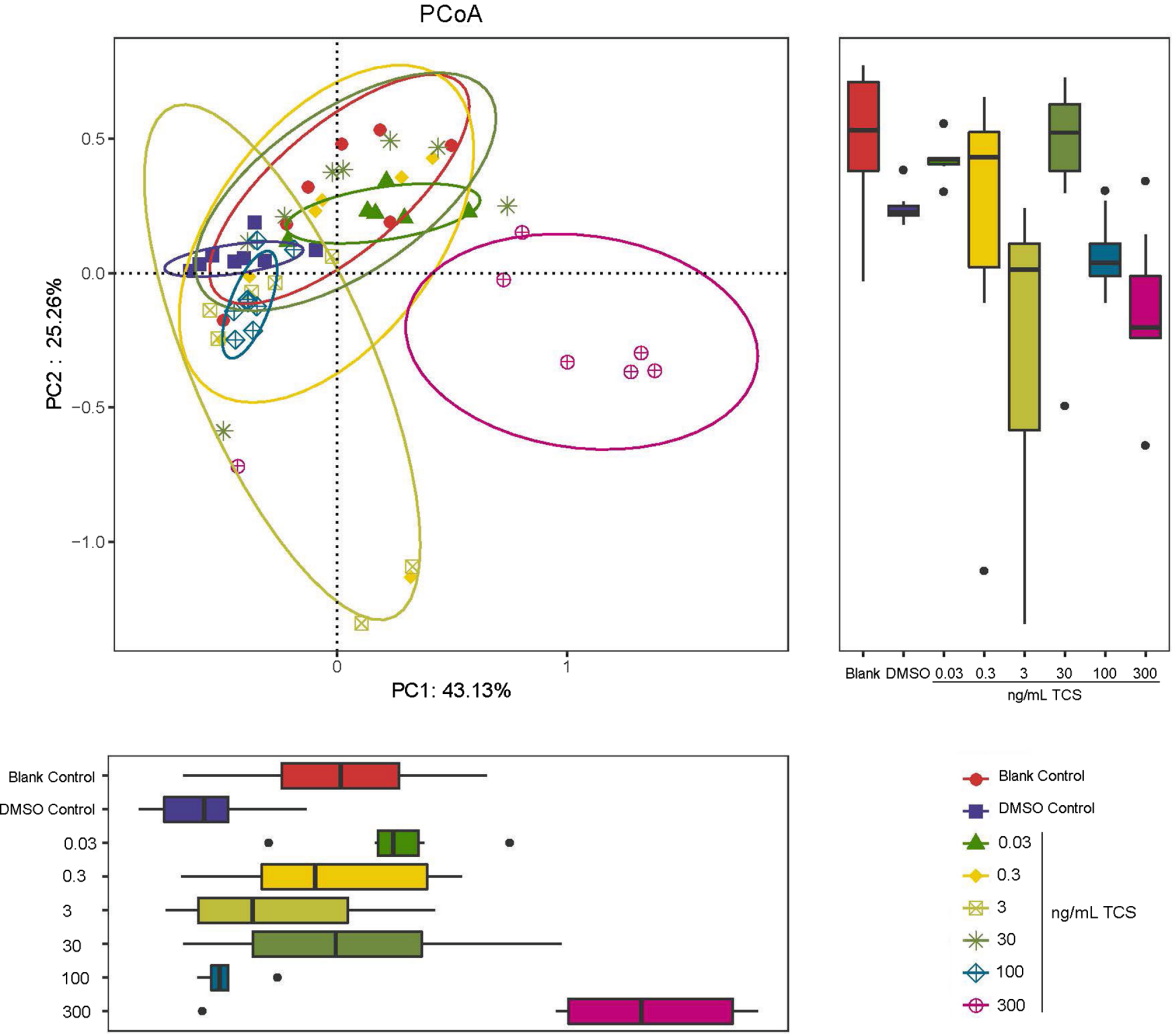
PCoA analysis of beta-diversity of microbiota in the gastrointestinal tract in zebrafish with different concentrations of TCS exposure. The bacterial communities of microbiota in the gastrointestinal tract in zebrafish clustered by TCS treatment on the PCoA diagram. TCS exposure at 300ng/ml level was associated with separation on PC1 and PC2.

PCoA diagram results showed that the zebrafish microbial community at 300ng/ml TCS exposure level clustered differently from those in DMSO control and other TCS exposure groups (Figure 2). Our analysis suggested associations between a high level of TCS exposure and altered microbiota composition.

### The Effect of Long-Term TCS Exposure on the Composition of Microbiota in the Gastrointestinal Tract

**Figure 3:**
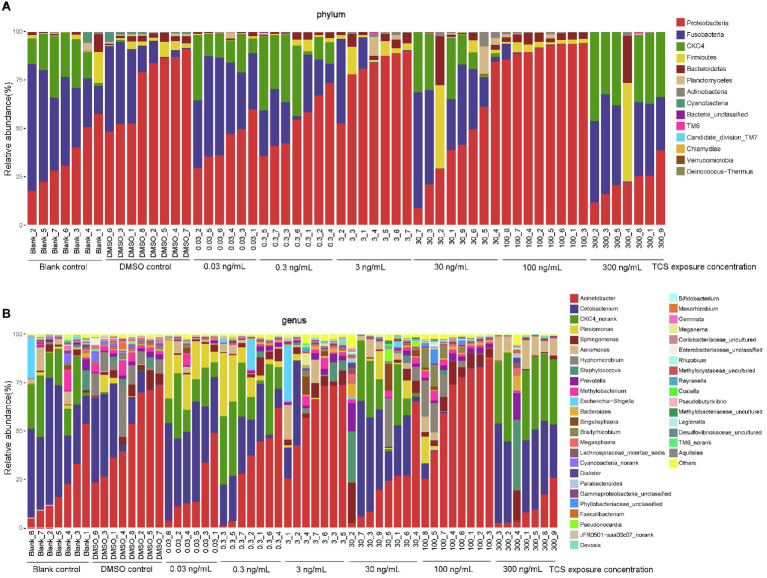
Composition of microbiota in zebrafish gastrointestinal tracts with different TCS exposure concentration at phylum **(A)** and genus **(B)** levels.

At the phylum level, the top 8 dominant microbiota phyla counted for 99.9% of microbiota in all zebrafish: *Proteobacteria* (mean 55.9%; range 8.9–94.0%)*, Fusobacteria* (23.0%; 0.004–65.6%)*, CKC4* (12.6%; 0.003–46.1%)*, Firmicutes* (3.7%; 0.03–50.9%)*, Bacteroidetes* (2.9%; 0.02–25.1%)*, Planctomycetes* (0.9%; 0–14.2%)*, Actinobacteria* (0.6%; 0.003–7.5%)*, Cyanobacteria* (0.3%; 0–5.5%; Figure 3A).

At the genus level, the top 8 dominant microbiota genera counted for 87.1% of microbiota in all zebrafish: *Acinetobacter* (mean 35.9%; range 1.3–87.7%), *Cetobacterium* (23.0%; 0.004–65.6%), *CKC4_norank* (12.6%; 0.003–46.1%), *Plesiomonas* (4.2%; 0–32.7%), *Sphingomonas* (3.6%; 0.03–20.9%), *Aeromonas* (3.1%; 0–17.9%), *Hyphomicrobium* (2.9%; 0–27.0%), *Staphylococcus* (1.9%; 0–36.6%; Figure 3B).

At the phylum level (Table 1), compared with the DMSO control, the relative abundance of *Proteobacteria* decreased in 0.03, 0.3, 30, and 300ng/ml TCS exposure groups, and increased in 100ng/ml TCS group; *Fusobacteria* increased in TCS 0.03ng/ml while decreased in 100ng/ml exposure group. The relative abundance of *CKC4* increased in TCS 0.03, 0.3, 30, and 300ng/ml groups; *Actinobacteria* proportion increased in 30ng/ml TCS exposure group; *Cyanobacteria* decreased in all TCS exposure groups.

**Table 1:** Effects of long-term TCS exposure on the relative abundance of the top 8 dominant microbiota in the gastrointestinal tract of zebrafish at phylum level.

		*Proteobacteria*		*Fusobacteria*		*CKC4*		*Firmicutes*	
TCS exposure levels (ng/mL)	*n*	mean±SD	*β*(95%CI)	mean±SD	*β*(95%CI)	mean±SD	*β*(95%CI)	mean±SD	*β*(95%CI)
0(DMSO)	8	0.7254 ± 0.1819	Reference	0.1934 ± 0.1920	Reference	0.0013 ± 0.0033	Reference	0.0298 ± 0.025	Reference
blank control	7	0.3535 ± 0.1468	−0.3719(−0.5254, −0.2185)^***^	0.3957 ± 0.1810	0.2023(0.0397, 0.3649)^*^	0.1819 ± 0.0978	0.1805(0.0771, 0.2840)^**^	0.0303 ± 0.0575	0.0004(−0.0948, 0.0957)
0.03	6	0.4293 ± 0.1122	−0.2961(−0.4562, −0.1360)^**^	0.3873 ± 0.1014	0.1939(0.0242, 0.3636)^*^	0.1616 ± 0.0948	0.1602(0.0523, 0.2682)^**^	0.0044 ± 0.0037	−0.0254(−0.1248, 0.0740)
0.3	7	0.5316 ± 0.1417	−0.1938(−0.3472, −0.0404)^*^	0.1921 ± 0.1019	−0.0014(−0.1640, 0.1612)	0.2321 ± 0.1474	0.2307(0.1272, 0.3342)^***^	0.0183 ± 0.0209	−0.0116(−0.1068, 0.0837)
3	7	0.8024 ± 0.1298	0.0770(−0.0764, 0.2304)	0.0865 ± 0.1659	−0.1070(−0.2696, 0.0557)	0.00037 ± 0.00063	−0.0010(−0.1044, 0.1025)	0.0457 ± 0.0478	0.0159(−0.0794, 0.1111)
30	8	0.4182 ± 0.2373	−0.3072(−0.4554, −0.1590)^**^	0.2812 ± 0.2147	0.0878(−0.0693, 0.2449)	0.1495 ± 0.1395	0.1481(0.0482, 0.2481)^**^	0.0654 ± 0.1494	0.0356(−0.0564, 0.1276)
100	8	0.9111 ± 0.0301	0.1857(0.0375, 0.3339)^*^	0.0124 ± 0.0279	−0.1810(−0.3381, −0.0239)^*^	0.00034 ± 0.0003	−0.0010(−0.1010, 0.0990)	0.0257 ± 0.0141	−0.0042(−0.0962, 0.0879)
300	7	0.2290 ± 0.0850	−0.4964(−0.6498, −0.3429)^***^	0.3402 ± 0.1644	0.1468(−0.0158, 0.3094)	0.3149 ± 0.1451	0.3135(0.2101, 0.4170)^***^	0.0734 ± 0.1922	0.0436(−0.0517, 0.1388)
*P*-trend^§^	0.18			0.66		0.04		0.30	
		***Bacteroidetes***		***Planctomycetes***		***Actinobacteria***		***Cyanobacteria***	
**TCS exposure levels (ng/mL)**	***n***	**mean±SD**	***β*(95%CI)**	**mean±SD**	***β*(95%CI)**	**mean±SD**	***β*(95%CI)**	**mean±SD**	***β*(95%CI)**
0(DMSO)	8	0.0262 ± 0.0119	Reference	0.008 ± 0.0043	Reference	0.0033 ± 0.0013	Reference	0.0124±0.0160	Reference
blank control	7	0.0201 ± 0.0318	−0.0062(−0.0557, 0.0434)	0.0071 ± 0.0156	−0.0009(−0.0253, 0.0235)	0.0026 ± 0.0043	−0.0007(−0.0117, 0.0103)	0.0086±0.0204	−0.0038(−0.0134, 0.0059)
0.03	6	0.0086 ± 0.0068	−0.0176(−0.0693, 0.0341)	0.0054 ± 0.0128	−0.0026(−0.0281, 0.0228)	0.0009 ± 0.0007	−0.0023(−0.0138, 0.0091)	3×10^−6^ ±8.16×10^−6^	−0.0124(−0.0224, −0.0023)^*^
0.3	7	0.02 ± 0.01470	−0.0062(−0.0558, 0.0434)	0.002 ± 0.0039	−0.0060(−0.0304, 0.0184)	0.0019 ± 0.0016	−0.0014(−0.0124, 0.0096)	0	−0.0124(−0.0220, −0.0028)^*^
3	7	0.037 ± 0.0207	0.0107(−0.0388, 0.0603)	0.0182 ± 0.0359	0.0102(−0.0142, 0.0346)	0.0083 ± 0.0070	0.0050(−0.0060, 0.0160)	9×10^−6^ ±2.46×10^−5^	−0.0124(−0.0220, −0.0028)^*^
30	8	0.0429 ± 0.0851	0.0167(−0.0312, 0.0646)	0.0235 ± 0.0490	0.0155(−0.0081, −0.0390)	0.0187 ± 0.0259	0.0154(0.0048, 0.0026)^**^	0	−0.0124(−0.0217, −0.0031)^*^
100	8	0.0373 ± 0.0131	0.0111(−0.0368, 0.0589)	0.0054 ± 0.0080	−0.0026(−0.0261, 0.0210)	0.0077 ± 0.0045	0.0044(−0.0062, 0.0150)	0	−0.0124(−0.0217, −0.0031)^*^
300	7	0.0355 ± 0.0918	0.0093(−0.0403, 0.0589)	0.0015 ± 0.0023	−0.0065(−0.0309, 0.0179)	0.0031 ± 0.0075	−0.0001 (−0.0111, 0.0109)	0	−0.0124(−0.0220, −0.0028)^*^
*P*-trend^§^	0.31			0.94		0.20		0.001	

§ *P-trend for DMSO control and TCS concentrations from 0.03–300ng/ml*.

* *P<0.0;*

** *p<0.01*

*** *p<0.0001*.

At the genus level (Table 2), compared with the DMSO control, the relative abundance of *Acinetobacter* decreased in TCS 0.03, 30, 300ng/ml; *Cetobacterium* increased in TCS 0.03ng/ml and decreased in 100ng/ml group; *CKC4_norank* increased in TCS 0.03, 0.3, 30, and 300ng/ml exposure groups; *Plesiomonas* increased in TCS 0.03 and 0.3ng/ml; *Hyphomicrobium* decreased in 0.03, 0.3, 3, 30, and 300ng/ml exposure groups; *Aeromonas* increased in TCS 3 and 300ng/ml exposure groups.

**Table 2:** Effects of long-term TCS exposure on the relative abundance of the top 8 dominant microbiota in the gastrointestinal tract of zebrafish at the genus level.

		*Acinetobacter*		*Cetobacterium*		*CKC4_norank*		*Plesiomonas*	
TCS exposure levels (ng/mL)	n	mean±SD	β (95%CI)	mean±SD	β (95%CI)	mean±SD	β (95%CI)	mean±SD	β (95%CI)
0 (DMSO)	8	0.4927 ± 0.2062	Reference	0.1934 ± 0.1920	Reference	0.0013 ± 0.0033	Reference	0.0155 ± 0.0313	Reference
blank control	7	0.2165 ± 0.1688	−0.2762(−0.4744, 0.0780)^**^	0.3956 ± 0.1810	0.2022(0.0396, 0.3648)^*^	0.1819 ± 0.0978	0.1805(0.0771, 0.2840)^**^	0.0079 ± 0.0078	−0.0076(−0.0679, 0.0527)
0.03	6	0.2056 ± 0.1712	−0.2870(−0.4939, −0.0802)^**^	0.3873 ± 0.1013	0.1939(0.0242, 0.3636)^*^	0.1616 ± 0.0948	0.1602(0.0523, 0.2682)^**^	0.1586 ± 0.0875	0.1431(0.0802, 0.2060)^***^
0.3	7	0.3181 ± 0.2258	−0.1745(−0.3727, 0.0237)	0.1919 ± 0.1020	−0.0015(−0.1641, 0.1611)	0.2321 ± 0.1474	0.2307(0.1272, 0.3342)^***^	0.1186 ± 0.1189	0.1031(0.0428, 0.1634)^**^
3	7	0.5881 ± 0.1862	0.0955(−0.1027, 0.2937)	0.0865 ± 0.1659	−0.1070(−0.2696, 0.0557)	0.0004 ± 0.0006	−0.0010(−0.1044, 0.1025)	0.0071 ± 0.0119	−0.0085(−0.0688, 0.0518)
30	8	0.2245 ± 0.1990	−0.2682(−0.496, −0.0767)^**^	0.2812 ± 0.2147	0.0878(−0.0694, 0.2449)	0.1495 ± 0.1395	0.1481(0.0482, 0.24481)^**^	0.0338 ± 0.0551	0.0183(−0.0400, 0.0765)
100	8	0.6613 ± 0.2284	0.1686(−0.0229, 0.3601)	0.0124 ± 0.0279	−0.1810(−0.3381, −0.0239)^*^	0.0003 ± 0.0003	−0.0010(−0.1010, 0.0990)	0.0202 ± 0.0476	0.0046(−0.05436, 0.0629)
300	7	0.0983 ± 0.0879	−0.3943(−0.5925, −0.1961)^**^	0.3400 ± 0.1644	0.1466(−0.0160, 0.3093)	0.3149 ± 0.1451	0.3135(0.2101, 0.4170)^***^	0.0019 ± 0.0027	−0.0136(−0.0740, 0.0467)
*P*-trend^§^		0.40		0.66		0.04		0.07	
		***Hyphomicrobium***		***Sphingomonas***		***Aeromonas***		***Staphylococcus***	
**TCS exposure levels (ng/mL)**	**n**	**mean±SD**	**β (95%CI)**	**mean±SD**	**β (95%CI)**	**mean±SD**	**β (95%CI)**	**mean±SD**	**β (95%CI)**
0 (DMSO)	8	0.0982 ± 0.0762	Reference	0.0422 ± 0.0282	Reference	0.0011 ± 0.0016	Reference	0.0130 ± 0.0191	Reference
blank control	7	0.0003 ± 0.0007	−0.0979(−0.1522, −0.0436)^**^	0.0150 ± 0.0149	−0.0271 (−0.0684, 0.0141)	0.0371 ± 0.0442	0.0360 (−0.0046, 0.0766)	0.0178 ± 0.0374	0.0047 (−0.0602, 0.0697)
0.03	6	0.0004 ± 0.0008	−0.0979(−0.1545, −0.0412)^**^	0.0121 ± 0.0123	−0.0301 (−0.0732, 0.0130)	0.0220 ± 0.0227	0.0209 (−0.0215, 0.0632)	0.0001 ± 0.0002	−0.0129 (−0.0807, 0.0549)
0.3	7	0.0002 ± 0.0002	−0.0981(−0.1524, −0.0438)^**^	0.0348 ± 0.0333	−0.0073 (−0.0486, 0.0339)	0.0180 ± 0.0201	0.0170 (−0.0236, 0.0576)	0.0045 ± 0.0117	−0.0086 (−0.0735, 0.0564)
3	7	0.0054 ± 0.0102	−0.0928(−0.1471, −0.0385)^**^	0.0433 ± 0.0243	0.0012 (−0.0401, 0.0424)	0.0445 ± 0.0739	0.0435 (0.0029, 0.0841)^*^	0.0235 ± 0.0383	0.0105 (−0.0545, 0.0754)
30	8	0.0443 ± 0.0874	−0.053 (−0.1064, −0.0014)^*^	0.0454 ± 0.0706	0.0032 (−0.0366, 0.0431)	0.0267 ± 0.0311	0.0256 (−0.0137, 0.0648)	0.0378 ± 0.0923	0.0247 (−0.0380, 0.0875)
100	8	0.0611 ± 0.0771	−0.0372(−0.0896, 0.0153)	0.0612 ± 0.0307	0.0190 (−0.0208, 0.0589)	0.0150 ± 0.0292	0.0139 (−0.0253, 0.0531)	0.0034 ± 0.0091	−0.0096 (−0.0724, 0.0532)
300	7	0.0003 ± 0.0004	−0.0979(−0.1522, −0.0436)^**^	0.0274 ± 0.0569	−0.0147 (−0.0560, 0.0265)	0.0854 ± 0.0483	0.0844 (0.0438, 0.1250)^**^	0.0523 ± 0.1381	0.0392 (−0.0257, 0.1042)
*P*-trend^§^		0.12		0.54		0.004		0.28	

§ *P-trend for DMSO control and TCS concentrations from 0.03–300ng/ml*.

* *P<0.05;*

** *P<0.01 and*

*** *P<0.0001*.

**Figure 4:**
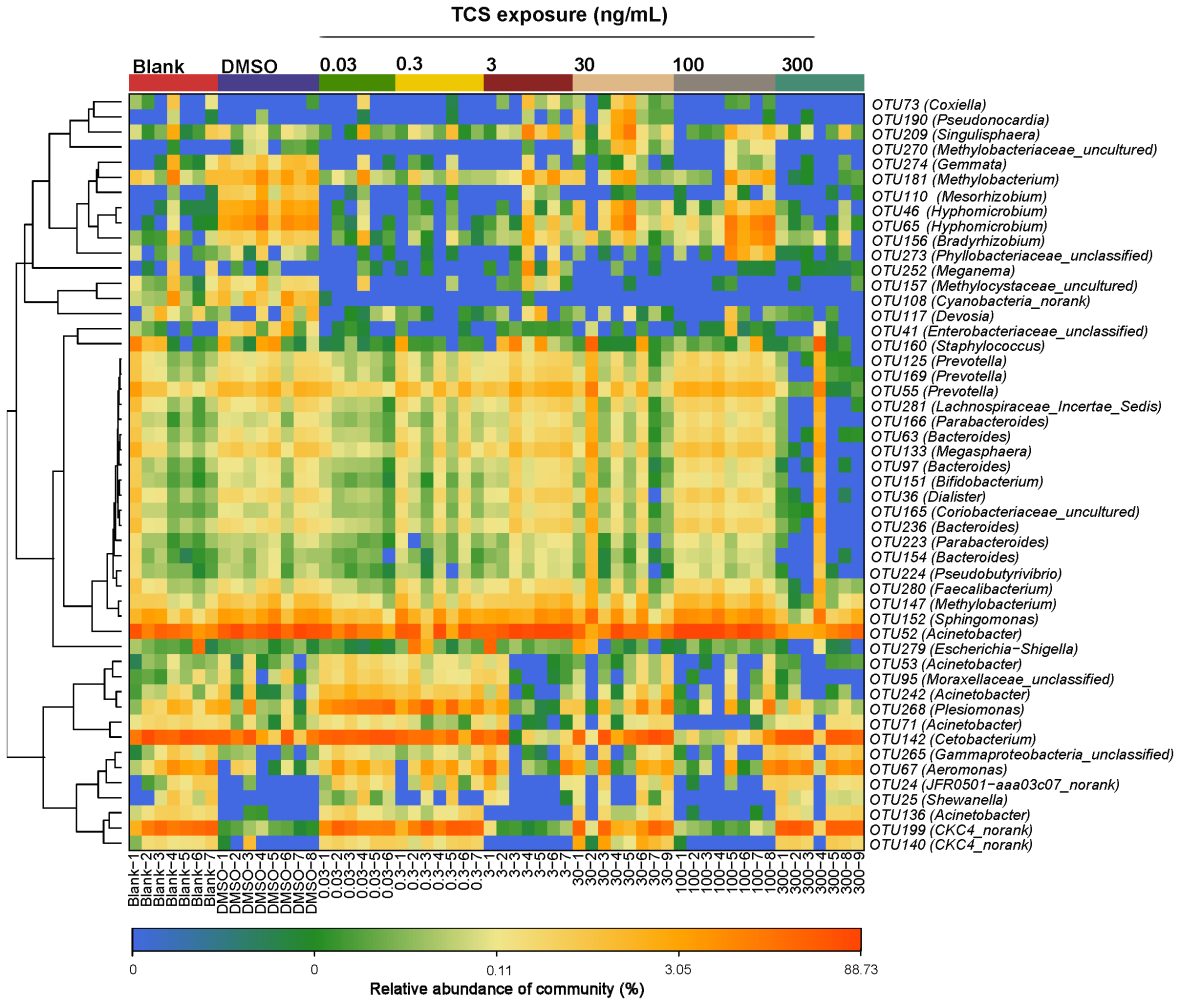
Top 50 OTU heatmap of bacteria in the gastrointestinal tract in zebrafish with different concentrations of TCS exposure.

At the OTUs level, the relative abundance of *Moraxellaceae_unclassified*, *Acinetobacter*, *Enterobacteriaceae_unclassified*, *Cyanobacteria_norank*, *Methylocystaceae_uncultured*, *Hyphomi crobium*, *Mesorhizobium*, *Methylobacterium*, *Gemmata* were lower in the highest TCS exposure group (300ng/ml) than the DMSO control group (Figure 4).

**Figure 5:**
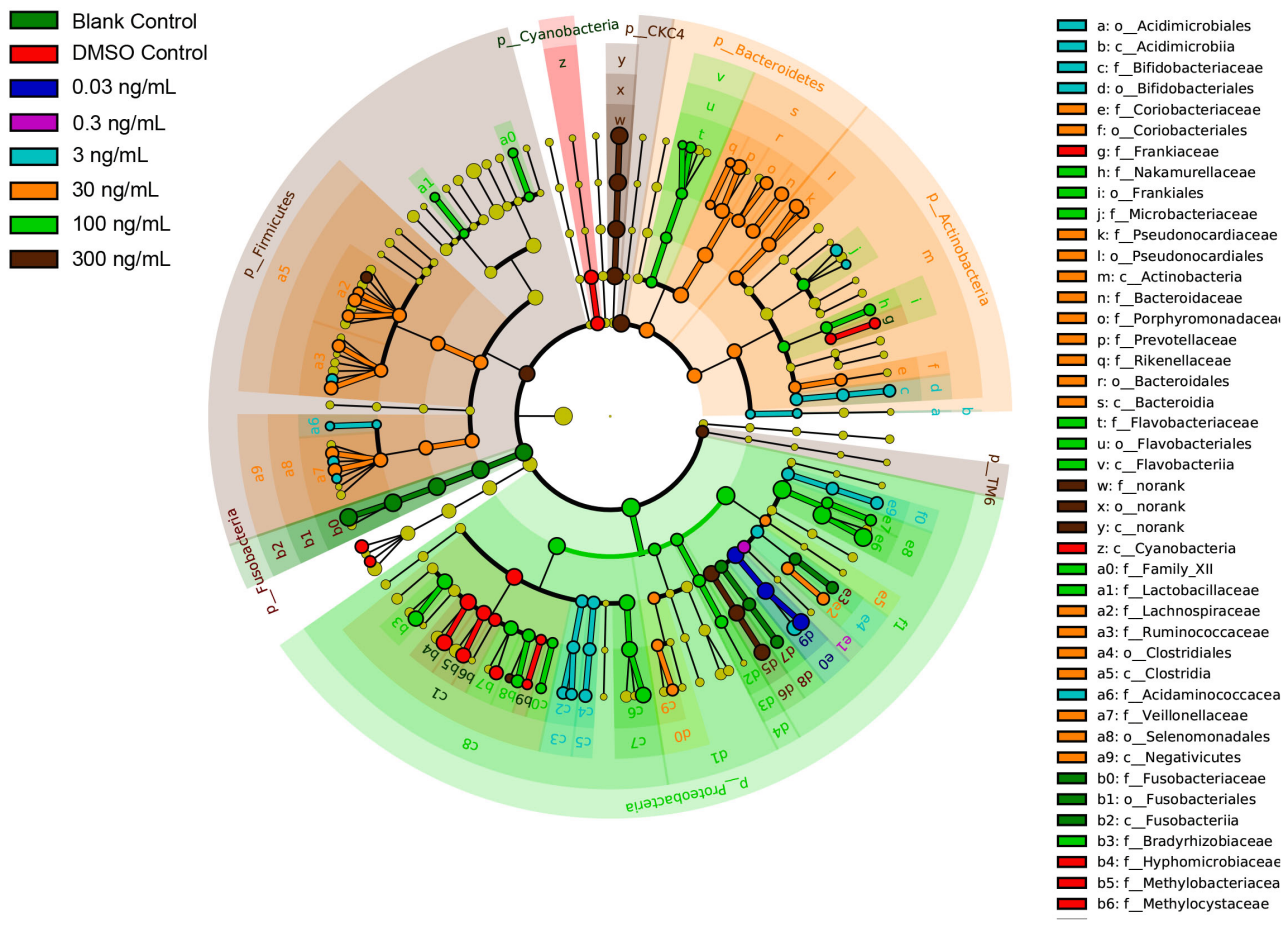
LDA Effect Size (LEfSe) cladograms of pairwise analysis for long-term TCS exposure with 16S rDNA sequence analysis in microbiota within the gastrointestinal tract of zebrafish. The cladogram shows the taxonomic levels represented by rings with phyla at the innermost ring and genera at the outermost ring, and each circle is a member within that level. Different taxa at class, order, and family levels are listed on the right side of the cladogram.

To further characterize the impact of TCS on the zebrafish microbiome in the gastrointestinal tract, discriminative features cladograms based on an effect size cutoff of 2.0 for the LDA score were plotted (Figure 5). Zebrafish at TCS 300ng/ml exposure exhibited higher relative abundances of phylum *Firmicutes*, *CKC4*, *TM6*, family *Aeromonadales*, order *Aeromonadaceae*, and genus *Aeromonas*, *Kaistia*, *Blautia*.

### Effects of Long-Term TCS Exposure on Body Length and Weight of Zebrafish

The effects of TCS exposure for 120days on the weight and body length of zebrafish are presented in Table 3. The body length of zebrafish was on average 1.90, 2.57, 1.92mm shorter at 0.03, 100, and 300ng/ml TCS, respectively, compared to that of the DMSO control group (*p*<0.05), but no difference was found in other TCS exposure levels (Table 3). There were no associations between TCS exposure and body weight (Table 3).

**Table 3:** The effects of different concentrations of TCS exposure on the weight and length of zebrafish.

Treatment		Weight (g)		Length (mm)	
TCS exposure levels (ng/mL)	n	mean±SD	β (95% CI)^#^	mean±SD	*β* (95% CI)^#^
0 (DMSO control)	8	0.22 ± 0.04	Reference	32.55 ± 1.12	Reference
blank control	7	0.25 ± 0.05	0.02(−0.03, 0.06)	31.80 ± 0.88	−0.74(−2.51, 1.03)
0.03	6	0.19 ± 0.04	−0.03(−0.07, 0.01)	30.65 ± 1.56	−1.90(−3.74, −0.07)^*^
0.3	7	0.22 ± 0.03	−0.01(−0.05, 0.04)	31.43 ± 0.85	−1.12(−2.88, 0.64)
3	7	0.22 ± 0.07	0.01(−0.04, 0.05)	31.34 ± 2.23	−1.21(−2.98, 0.56)
30	8	0.20 ± 0.02	−0.02(−0.06, 0.02)	31.18 ± 1.04	−1.38(−3.08, 0.32)
100	8	0.21 ± 0.05	−0.01(−0.06, 0.03)	29.98 ± 3.12	−2.58(−4.27, −0.88)^**^
300	7	0.19 ± 0.02	−0.03(−0.08, 0.01)	30.63 ± 0.90	−1.92(−3.68, −0.16)^*^
*P*-trend^§^			0.21		0.02

§ *P-trend for DMSO control and TCS concentrations from 0.03–300ng/ml*.

# *all models were adjusted for sex*.

* *P<0.05 and*

** *p<0.01*.

### Short-term 7-Day Exposure to TCS and Microbiota in the Gastrointestinal Tract of Adult Zebrafish

We examined whether short-term TCS exposure affected microbiota in adult zebrafish that grew without TCS until 4months post hatching, and then was given TCS for 7days.

In short-term TCS exposure, there were no differences in alpha-diversity (OTU numbers and Chao indices, (Supplementary Figure S2) or beta-diversity (Supplementary Figure S3) of microbiota in adult zebrafish between TCS exposure groups and DMSO control. Similarly, phylum *Proteobacteria, Fusobacteria, Actinobacteria, Firmicutes, Bacteroidetes*, and *Cyanobacteria* accounted for 95.6% of microbiota (Supplementary Figure S4A). The relative abundance of the top 2 most dominant phyla, *Proteobacteria*, and *Fusobacteria* accounted for 90.5% and showed no difference among the DMSO control group and all TCS exposure groups (Supplementary Table S1). Compared with the DMSO control, the relative abundance of phylum *Tenericutes* was higher in the TCS 0.03ng/ml group. The relative abundance of *Actinobacteria* was higher in TCS 100 and 300ng/ml groups; *Firmicutes* was lower in 100 and 900ng/ml exposure groups; *Bacteria_unclassified* was lower in all TCS exposure groups (Supplementary Table S1).

The top 8 dominant genera accounted for 90.0% of microbiota in all zebrafish (Supplementary Figure S4B, Supplementary Table S2), among which the three genera *Cetobacterium*, *Aeromonas*, and *Plesiomonas* accounted for 49.1% (Supplementary Figure S4B), and showed no difference among DMSO control and TCS exposure groups. Compared with DMSO control, the relative abundance of genus *Shinella* was higher in TCS 30, 100, 300ng/ml groups; the *Mycoplasma* proportion was higher in the 0.03ng/ml TCS group. The relative abundance of *Arthrobacter* was higher in TCS 300 and 900ng/ml groups, while the relative abundance of *Bosea* was lower in the 900ng/ml TCS group (Supplementary Table S2).

### Replicate Experiment on the Effect of Long-Term High TCS Exposure

In addition, we conducted a replicate experiment to compare the effect between long-term exposure to TCS 300ng/ml and DMSO control starting from the time of fertilized zebrafish eggs to adulthood (75 dpf). The alpha-diversity (number of observed OTU) was lower for gastrointestinal microbiota of zebrafishes exposed to TCS 300ng/ml, and the beta-diversity of microbiota differed between the TCS 300ng/ml and DMSO control groups (Supplementary Figures S5, S6). The top two most dominant microbiota phyla were *Fusobacteria* and *Proteobacteria* phylum in all zebrafishes (data not shown). In the replicate experiment, a shorter body length of zebrafish was observed in long-term TCS exposure at 300ng/ml compared to that of the DMSO control group (mean±SD: 27±1 vs. 25±3mm, *p*=0.04).

## Discussion

In this study, we explored the effects of long-term exposure to TCS, during the first 120days of life, on microbiota in the gastrointestinal tract using a zebrafish model. The original contribution of this study is the finding that the TCS exposure window spanned the entire period from early embryonic life to early adulthood. We found that long-term exposure to high TCS concentration (300ng/ml) reduced alpha-diversity, and altered the composition of microbiota, with a decrease in *Proteobacteria*, the disappearance of *Cyanobacteria*, and enrichment in *CKC4* at *the* phylum level, and the body length of zebrafish was slightly shorter. We also found that the diversity of microbiota in zebrafish adults with short-term exposure was not affected by any TCS, but a minor change in microbial composition was observed in high TCS exposure.

Our study found that TCS exposure was associated with decreased richness and alpha-diversity, and altered relative abundance of microbiota phylum in the gastrointestinal tract of zebrafish. Our results were consistent with the only one available study focused on the effects of short-term TCS exposure on the gut microbiota of zebrafish until now (Gaulke et al., 2016), it showed that acute TCS exposure for 4 or 7days changed gut microbial community structure and reduced Shannon indices and richness in adult zebrafish (Gaulke et al., 2016). However, a study conducted in fathead minnow showed that the differences of both alpha and beta-diversity of gut microbial communities caused by 7-day TCS exposure disappeared after two weeks of depuration (Narrowe et al., 2015). Rodent studies found that TCS exposure changed the relative abundance of microbiota on family level or the intestinal flora balance in mice (Gao et al., 2017; Jin et al., 2018), decreased the alpha-diversity, and resulted in gut bacteria assemblages with distinct trajectories compared to controls in rats (Hu et al., 2016). In human studies, the gut microbiomes of infants who received breast milk containing TCS had significantly lower Shannon diversity and differential abundant taxa compared with the infants who received breast milk with non-detectable levels of TCS (Bever et al., 2018). Different duration of exposure, the maturation extent of gut microbiota, and exposure route may partially explain the different findings among these studies (Lawrence et al., 2015; Gaulke et al., 2016). Our results provide a better understanding of the effect of long-term TCS exposure on microbiota in the gastrointestinal tract.

The relationship between the toxic effects of TCS and the induced structural disturbance of intestinal flora has not been demonstrated. After TCS enters the body, it is rapidly removed through the kidney or gastrointestinal tract by metabolizing water-soluble conjugates (e.g., TCS glucuronide and TCS sulfate). While in the colon tissues, the beta-glucuronidase- and sulfatase-expressing gut bacteria could catalyze the hydrolysis of TCS glucuronide and TCS sulfate, and regenerate the TCS (Pellock and Redinbo, 2017). Therefore, TCS could be inversely metabolized in colon tissues, which may contribute to its toxic effect on the gut. In addition, Toll-like receptor 4 (TLR4), an innate immunity receptor that plays critical roles in recognizing gut bacteria, may mediate the adverse effects of TCS on gut microbiota (Yang et al., 2018).

In our study, the significantly altered relative abundance of microbiota in the gastrointestinal tract of zebrafish was observed only at 300ng/ml TCS level which was about three-fold the upper limit of human urine TCS level (Wang et al., 2017), indicating the potential health effect of high TCS exposure level on microbiota in the gastrointestinal tract. No significant change was observed in the diversity of microbiota in the gastrointestinal tract of adult zebrafish when exposed to TCS for only 7days. Consistently, in a rat study, increased abundance was observed for *Deltaproteobacteria* in the TCS group only in adolescence, but not in adulthood (Hu et al., 2016). One study also found that 7-day exposure to TCS altered the gut microbiome in fathead minnow larvae exposed to TCS 100ng/l and 1,000ng/l solution (Narrowe et al., 2015). However, another study in adult zebrafish showed that 7-day exposure to TCS changed the composition of gut microbiota when exposed to a diet of 100μg/g TCS (Gaulke et al., 2016), which may support the idea that gut microbiota diversity decreases with the course of development and remains relatively stable in the adult period (Stephens et al., 2016).

Both in long-term and short-term experiments, our study found that the top dominant phyla were *Proteobacteria*, *Fusobacteria*, and *Firmicutes* for the microbiome in the gastrointestinal tract of zebrafish. This was consistent with studies of gut microbiota in zebrafish (Stephens et al., 2016). *Proteobacteria* and *Fusobacteria* were persistently identified as the most dominant phyla, despite the increasing interindividual and intraindividual variation with age (Stephens et al., 2016). In addition, the core gut microbiota of zebrafish (*Proteobacteria*, *Fusobacteria*, and *Firmicutes*) was stable in nature or laboratory environment though the difference in bred and raised conditions (salinity and housing environmental system; Roeselers et al., 2011, Breen et al., 2019).

In our study, at the phylum level, zebrafish exposed to 300ng/ml TCS for 120days had a lower relative abundance of *Proteobacteria*. Another study also showed that TCS exposure can reduce the relative abundance of *Proteobacteria* in zebrafish, though the exposure duration was less than 7days (Gaulke et al., 2016). Conversely, in a human study, a strong enrichment of *Proteobacteria* in gut microbiota was observed in mothers after the introduction of TCS-containing toothpaste and in infants with higher urine TCS levels (Ribado et al., 2017). *Proteobacteria* enrichment has been indirectly associated with increased antibiotic resistance genes in the human gut microbiota and may be used as a marker for community antibiotic resistance (Bengtsson-Palme et al., 2015). The *Proteobacteria* phylum contains both pathogenic and nonpathogenic species, which makes it difficult to ascribe clinical or public health relevance to our present results (Ribado et al., 2017).

This study showed a higher relative abundance of phylum *CKC4* in the zebrafish exposed to 300ng/ml TCS compared to the DMSO control. One previous study also found that zebrafish exposed to Bisphenol A (BPA) and estrogen (E_2_) had significantly increased abundance of the phylum *CKC4* (Yin et al., 2016). There are limited functional studies of *CKC4*, a phylum in the SLIVA database. Taken together, we cannot exclude the possibility that the rise of the proportion of phylum *CKC4* may be sensitive to EDCs.

In our study, the relative abundance of genus *Staphylococcus* in microbiota was higher in zebrafish exposed to TCS at 300ng/ml group, when compared to the DMSO control group. There are increasing concerns that TCS-containing products contribute to the development of antibiotic resistance. Some *Staphylococcus* strains were found resistant to TCS (Suller and Russell, 2000). A previous lab study showed that exposure to TCS at a low concentration (0.0004%) was associated with a high risk of developing resistance to ampicillin and/or ciprofloxacin in *Staphylococcus aureus* (Wesgate et al., 2016). In a human study, 5-year use of TCS-containing toothpaste was not found to be associated with TCS-resistant bacterial population including *Staphylococcus* in dental plaque (Cullinan et al., 2014). However, an animal study showed that *Staphylococcus aureus* was more likely to colonize in noses in TCS exposed rats compared to the placebo group (Syed et al., 2014).

This study found that the highest TCS concentration (300ng/ml) altered the microbiome composition of zebrafish. These associations were not observed in lower TCS concentration exposure, the level commonly detected in rivers and lakes (Coogan et al., 2007; Lyndall et al., 2010). However, TCS can be bio-accumulated in fish and other species (Arnot et al., 2018), further study in natural conditions (i.e., river and lakes) is needed.

Because TCS has low water solubility, DMSO is commonly added to dissolve TCS in animal studies (Nassef et al., 2010; Zhou et al., 2017). Previous studies reported that DMSO concentrations up to 10% (v/v) did not affect the bacterial-inhibition ability of phenol compounds (Modrzynski et al., 2019), and DMSO concentration of 0.25% did not impede the viability of *E. coli* (Negin et al., 2014). In our study, DMSO concentration was set at a low concentration (0.01%) and the same in both DMSO control and all the TCS exposure solutions; However, in this study, there was a difference in the relative abundance of three phyla (*Proteobacteria*, *Fusobacteria*, and *CKC4*) and four genera (*Acinetobacter*, *Cetobacterium*, *CKC4_norank*, *Hyphomicrobium*) between the DMSO control and blank control groups. It is unknown whether DMSO may modify the association between TCS and the relative abundance of the above microbiota phyla and genera, caution should be paid in interpreting these results.

We found that the body length of fish was shorter in TCS 100 and 300ng/ml exposure. This is consistent with previous studies. It was found that high TCS exposure (600ng/ml) decreases body length in zebrafish embryos (Kim et al., 2018). The change of gut microbiota caused by high TCS exposure may have adverse effects on the growth of zebrafish.

There are some strengths and limitations to our study. The strengths of this study include a broad range of TCS concentrations and that it was carried out over a long period of time. First, concentrations of TCS used in our study were comparable to those in the environment and human bio-samples. Therefore, the present results provide a guarantee for greater ecological relevance. Second, a long-term continuous exposure model from the fertilized period may simulate the frequent exposure model from the embryo stage in animals and humans to a certain degree. The limitation of this study is the small sample size of different genders, and in future studies, it would be better to analyze the TCS effect on microbiota adjusted by gender. Second, we did not conduct longitudinal observation of the gut microbiota for a better understanding of the effect of TCS exposure on the trajectory of gut microbiota establishment and development in zebrafish. In this study, the zebrafish were raised in tanks with enough volume (1,000ml) of exposure medium per tank, and we changed half of the medium each time, twice per day. All glass tanks/containers were autoclaved prior to use. Thus the water environment was comparable between the tanks except for TCS levels. In the present study, the main finding was the lower microbial diversity in high TCS (300ng/ml) long-term exposure. Due to the antibacterial feature of TCS, this finding is biologically plausible. Consistent with previous research, this study found that the core gastrointestinal microbiota included bacterial phyla *Proteobacteria*, *Fusobacteria*, and *Firmicutes* (Roeselers et al., 2011). The composition of the gut microbial community is mainly shaped by selective pressures (Roeselers et al., 2011; Breen et al., 2019). However, patterns of gut microbiota structure may vary between different studies due to variation of season, water chemistry, diet composition, feeding schedule, or the housing infrastructure of different labs. In addition, the relative abundance (i.e., proportion), instead of absolute values were used to quantify gastrointestinal microbiota, which can be influenced not only by the amount of specific microbiota phylum and genus as numerator but also by the total numbers of phyla and genera microbiota as denominator. The lack of technical quantification of the absolute amount of microbiota might limit the detecting capacity of change of specific microbiota in the gastrointestinal tract caused by TCS exposure.

## Conclusion

In this study, we found that long-term exposure to high TCS concentration from early embryonic life to early adulthood may lower diversity and alter the composition of microbiota in the gastrointestinal tract of zebrafish. In adult zebrafish with short-term TCS exposure, no effect was observed on the diversity of microbiota in any TCS exposure, but a minor difference in microbial composition was observed in TCS exposure.

## Data Availability Statement

The datasets presented in this study can be found in online repositories. The names of the repository/repositories and accession number(s) can be found at: https://www.ncbi.nlm.nih.gov/PRJNA682251, and https://www.ncbi.nlm.nih.gov/PRJNA759445.

## Ethics Statement

The animal study was reviewed and approved by Ethics Committee of Xinhua Hospital Affiliated to Shanghai Jiao Tong University School of Medicine (NO. XHEC-F-2018-059).

## Author Contributions

FO: conceptualization, design, data analysis and interpretation, writing - original draft and revision, and funding acquisition. NT: research conduction, data analysis, and writing - paper draft. PF: research conduction, data analysis and writing -revision. XY: research conduction. YT, RM, and WW: writing -review and revision. FO is the guarantor of this work and had full access to all data. All authors approved the submitted manuscript.

## Funding

This work was supported by grants from the National Natural Science Foundation of China (No. 81961128023, No. 81673178), the Ministry of Science and Technology of China (No. 2017YFE0124700), Shanghai Municipal Education Commission—Gaofeng Clinical Medicine Grant (20152518), and Shanghai Municipal Health Commission (2020CXJQ01, GWV-10.1-XK07).

## Conflict of Interest

The authors declare that the research was conducted in the absence of any commercial or financial relationships that could be construed as a potential conflict of interest.

## Publisher’s Note

All claims expressed in this article are solely those of the authors and do not necessarily represent those of their affiliated organizations, or those of the publisher, the editors and the reviewers. Any product that may be evaluated in this article, or claim that may be made by its manufacturer, is not guaranteed or endorsed by the publisher.

## Footnotes

^1^ https://www.illumina.com/systems/sequencing-platforms/

^2^ http://drive5.com/uparse/

^3^ http://rdp.cme.msu.edu/

^4^ http://www.R-project.org/

## References

[ref1] Amato K. R. Yeoman C. J. Kent A. Righini N. Carbonero F. Estrada A.. (2013). Habitat degradation impacts black howler monkey (Alouatta pigra) gastrointestinal microbiomes. ISME J. 7, 1344–1353. doi: 10.1038/ismej.2013.16, PMID: 23486247 PMC3695285

[ref2] Arnot J. A. Pawlowski S. Champ S. (2018). A weight-of-evidence approach for the bioaccumulation assessment of triclosan in aquatic species. Sci. Total Environ. 618, 1506–1518. doi: 10.1016/j.scitotenv.2017.09.322 29029804

[ref3] Balasubramani A. Pandian T. J. (2008). Endosulfan suppresses growth and reproduction in zebrafish. Curr. Sci. 94, 883–890. doi: 10.1126/science.1154228

[ref4] Bengtsson-Palme J. Angelin M. Huss M. Kjellqvist S. Kristiansson E. Palmgren H.. (2015). The human gut microbiome as a transporter of antibiotic resistance genes between continents. Antimicrob. Agents Chemother. 59, 6551–6560. doi: 10.1128/AAC.00933-15, PMID: 26259788 PMC4576037

[ref5] Bever C. S. Rand A. A. Nording M. Taft D. Kalanetra K. M. Mills D. A.. (2018). Effects of triclosan in breast milk on the infant fecal microbiome. Chemosphere 203, 467–473. doi: 10.1016/j.chemosphere.2018.03.186, PMID: 29635158 PMC5915298

[ref6] Breen P. Winters A. D. Nag D. Ahmad M. M. Theis K. R. Withey J. H. (2019). Internal versus external pressures: effect of housing systems on the zebrafish microbiome. Zebrafish 16, 388–400. doi: 10.1089/zeb.2018.1711, PMID: 31145047 PMC6685215

[ref7] Calafat A. M. Ye X. Wong L. Y. Reidy J. A. Needham L. L. (2008). Urinary concentrations of triclosan in the U.S. population: 2003-2004. Environ. Health Perspect. 116, 303–307. doi: 10.1289/ehp.10768, PMID: 18335095 PMC2265044

[ref8] Caporaso J. G. Lauber C. L. Walters W. A. Berg-Lyons D. Huntley J. Fierer N.. (2012). Ultra-high-throughput microbial community analysis on the Illumina HiSeq and MiSeq platforms. ISME J. 6, 1621–1624. doi: 10.1038/ismej.2012.8, PMID: 22402401 PMC3400413

[ref10] Coogan M. A. Edziyie R. E. La Point T. W. Venables B. J. (2007). Algal bioaccumulation of triclocarban, triclosan, and methyl-triclosan in a North Texas wastewater treatment plant receiving stream. Chemosphere 67, 1911–1918. doi: 10.1016/j.chemosphere.2006.12.027, PMID: 17275881

[ref11] Cullinan M. P. Bird P. S. Heng N. C. West M. J. Seymour G. J. (2014). No evidence of triclosan-resistant bacteria following long-term use of triclosan-containing toothpaste. J. Periodontal Res. 49, 220–225. doi: 10.1111/jre.12098, PMID: 23668824

[ref12] Dann A. B. Hontela A. (2011). Triclosan: environmental exposure, toxicity and mechanisms of action. J. Appl. Toxicol. 31, 285–311. doi: 10.1002/jat.1660, PMID: 21462230

[ref13] Edgar R. C. (2010). Search and clustering orders of magnitude faster than BLAST. Bioinformatics 26, 2460–2461. doi: 10.1093/bioinformatics/btq461, PMID: 20709691

[ref14] Gao B. Tu P. Bian X. Chi L. Ru H. Lu K. (2017). Profound perturbation induced by triclosan exposure in mouse gut microbiome: a less resilient microbial community with elevated antibiotic and metal resistomes. BMC Pharmacol. Toxicol. 18:46. doi: 10.1186/s40360-017-0150-9 28606169 PMC5469155

[ref15] Gaulke C. A. Barton C. L. Proffitt S. Tanguay R. L. Sharpton T. J. (2016). Triclosan exposure is associated with rapid restructuring of the microbiome in adult zebrafish. PLoS One 11:e0154632. doi: 10.1371/journal.pone.0154632, PMID: 27191725 PMC4871530

[ref16] Godon J. J. Zumstein E. Dabert P. Habouzit F. Moletta R. (1997). Molecular microbial diversity of an anaerobic digestor as determined by small-subunit rDNA sequence analysis. Appl. Environ. Microbiol. 63, 2802–2813. doi: 10.1128/aem.63.7.2802-2813.1997, PMID: 9212428 PMC168577

[ref17] Goldsmith P. (2004). Zebrafish as a pharmacological tool: the how, why and when. Curr. Opin. Pharmacol. 4, 504–512. doi: 10.1016/j.coph.2004.04.005, PMID: 15351356

[ref18] Halden R. U. (2014). On the need and speed of regulating triclosan and triclocarban in the United States. Environ. Sci. Technol. 48, 3603–3611. doi: 10.1021/es500495p, PMID: 24588513 PMC3974611

[ref19] Halden R. U. (2016). Lessons learned from probing for impacts of Triclosan and Triclocarban on human microbiomes. mSphere 1, e00089–e00016. doi: 10.1128/mSphere.00089-1 PMC4888884 27306705

[ref20] Hu J. Raikhel V. Gopalakrishnan K. Fernandez-Hernandez H. Lambertini L. Manservisi F.. (2016). Effect of postnatal low-dose exposure to environmental chemicals on the gut microbiome in a rodent model. Microbiome. 4:26. doi: 10.1186/s40168-016-0173-2 27301250 PMC4906585

[ref21] Jemielita M. Taormina M. J. Burns A. R. Hampton J. S. Rolig A. S. Guillemin K.. (2014). Spatial and temporal features of the growth of a bacterial species colonizing the zebrafish gut. MBio 5:e01751-14. doi: 10.1128/mBio.01751-14, PMID: 25516613 PMC4271548

[ref22] Jin Y. Xia J. Pan Z. Yang J. Wang W. Fu Z. (2018). Polystyrene microplastics induce microbiota dysbiosis and inflammation in the gut of adult zebrafish. Environ. Pollut. 235, 322–329. doi: 10.1016/j.envpol.2017.12.088, PMID: 29304465

[ref23] Kim J. Oh H. Ryu B. Kim U. Lee J. M. Jung C. R.. (2018). Triclosan affects axon formation in the neural development stages of zebrafish embryos (Danio rerio). Environ. Pollut. 236, 304–312. doi: 10.1016/j.envpol.2017.12.110, PMID: 29414352

[ref24] Kumar K. S. Priya S. M. Peck A. M. Sajwan K. S. (2010). Mass loadings of triclosan and triclocarbon from four wastewater treatment plants to three rivers and landfill in Savannah, Georgia. USA. Arch. Environ. Contam. Toxicol. 58, 275–285. doi: 10.1007/s00244-009-9383-y, PMID: 19756845

[ref25] Lantz-McPeak S. Guo X. Cuevas E. Dumas M. Newport G. D. Ali S. F.. (2015). Developmental toxicity assay using high content screening of zebrafish embryos. J. Appl. Toxicol. 35, 261–272. doi: 10.1002/jat.3029, PMID: 24871937 PMC4957247

[ref26] Lawrence J. R. Topp E. Waiser M. J. Tumber V. Roy J. Swerhone G. D.. (2015). Resilience and recovery: the effect of triclosan exposure timing during development, on the structure and function of river biofilm communities. Aquat. Toxicol. 161, 253–266. doi: 10.1016/j.aquatox.2015.02.012, PMID: 25731684

[ref27] Li X. Ying G. G. Su H. C. Yang X. B. Wang L. (2010). Simultaneous determination and assessment of 4-nonylphenol, bisphenol A and triclosan in tap water, bottled water and baby bottles. Environ. Int. 36, 557–562. doi: 10.1016/j.envint.2010.04.009, PMID: 20452023

[ref28] Lyndall J. Fuchsman P. Bock M. Barber T. Lauren D. Leigh K.. (2010). Probabilistic risk evaluation for triclosan in surface water, sediments, and aquatic biota tissues. Integr. Environ. Assess. Manag. 6, 419–440. doi: 10.1897/IEAM_2009-072.1, PMID: 20821705

[ref30] Modrzynski J. J. Christensen J. H. Brandt K. K. (2019). Evaluation of dimethyl sulfoxide (DMSO) as a co-solvent for toxicity testing of hydrophobic organic compounds. Ecotoxicology 28, 1136–1141. doi: 10.1007/s10646-019-02107-0, PMID: 31559559

[ref31] Nag S. K. Das Sarkar S. Manna S. K. (2018). Triclosan - an antibacterial compound in water, sediment and fish of river Gomti. India. Int. J. Environ. Health Res. 28, 461–470. doi: 10.1080/09603123.2018.1487044 29925273

[ref32] Narrowe A. B. Albuthi-Lantz M. Smith E. P. Bower K. J. Roane T. M. Vajda A. M.. (2015). Perturbation and restoration of the fathead minnow gut microbiome after low-level triclosan exposure. Microbiome. 3:6. doi: 10.1186/s40168-015-0069-6 25815185 PMC4374533

[ref33] Nassef M. Matsumoto S. Seki M. Khalil F. Kang I. J. Shimasaki Y.. (2010). Acute effects of triclosan, diclofenac and carbamazepine on feeding performance of Japanese medaka fish (Oryzias latipes). Chemosphere 80, 1095–1100. doi: 10.1016/j.chemosphere.2010.04.073, PMID: 20537681

[ref34] Negin S. Gokel M. R. Patel M. B. Sedinkin S. L. Osborna D. C. Gokel G. W. (2014). The aqueous medium-dimethylsulfoxide conundrum in biological studies. RSC Adv. 5, 8088–8093. doi: 10.1039/C4RA15217D

[ref35] Oliveira J. M. Almeida A. R. Pimentel T. Andrade T. S. Henriques J. F. Soares A. M.. (2016). Effect of chemical stress and ultraviolet radiation in the bacterial communities of zebrafish embryos. Environ. Pollut. 208, 626–636. doi: 10.1016/j.envpol.2015.10.039 26552525

[ref36] Oliveira R. Domingues I. Koppe Grisolia C. Soares A. M. (2009). Effects of triclosan on zebrafish early-life stages and adults. Environ. Sci. Pollut. Res. Int. 16, 679–688. doi: 10.1007/s11356-009-0119-3, PMID: 19283420

[ref37] Pellock S. J. Redinbo M. R. (2017). Glucuronides in the gut: sugar-driven symbioses between microbe and host. J. Biol. Chem. 292, 8569–8576. doi: 10.1074/jbc.R116.767434, PMID: 28389557 PMC5448086

[ref38] Peng X. Yu Y. Tang C. Tan J. Huang Q. Wang Z. (2008). Occurrence of steroid estrogens, endocrine-disrupting phenols, and acid pharmaceutical residues in urban riverine water of the Pearl River Delta. South China. Sci. Total Environ. 397, 158–166. doi: 10.1016/j.scitotenv.2008.02.059 18407320

[ref39] Philippat C. Mortamais M. Chevrier C. Petit C. Calafat A. M. Ye X.. (2012). Exposure to phthalates and phenols during pregnancy and offspring size at birth. Environ. Health Perspect. 120, 464–470. doi: 10.1289/ehp.1103634, PMID: 21900077 PMC3295340

[ref40] Ribado J. V. Ley C. Haggerty T. D. Tkachenko E. Bhatt A. S. Parsonnet J. (2017). Household triclosan and triclocarban effects on the infant and maternal microbiome. EMBO Mol. Med. 9, 1732–1741. doi: 10.15252/emmm.201707882, PMID: 29030459 PMC5709730

[ref41] Robertson R. C. Manges A. R. Finlay B. B. Prendergast A. J. (2019). The human microbiome and child growth – first 1000 days and Beyond. Trends Microbiol. 27, 1311–1347. doi: 10.1016/j.tim.2018.09.008 30529020

[ref42] Roeselers G. Mittge E. K. Stephens W. Z. Parichy D. M. Cavanaugh C. M. Guillemin K.. (2011). Evidence for a core gut microbiota in the zebrafish. ISME J. 5, 1595–1608. doi: 10.1038/ismej.2011.38, PMID: 21472014 PMC3176511

[ref43] Stephens W. Z. Burns A. R. Stagaman K. Wong S. Rawls J. F. Guillemin K.. (2016). The composition of the zebrafish intestinal microbial community varies across development. ISME J. 10, 644–654. doi: 10.1038/ismej.2015.140, PMID: 26339860 PMC4817687

[ref44] Stephens W. Z. Wiles T. J. Martinez E. S. Jemielita M. Burns A. R. Parthasarathy R.. (2015). Identification of population bottlenecks and colonization factors during assembly of bacterial communities within the zebrafish intestine. MBio 6, e01163–e01115. doi: 10.1128/mBio.01163-15, PMID: 26507229 PMC4626852

[ref45] Suller M. T. Russell A. D. (2000). Triclosan and antibiotic resistance in Staphylococcus aureus. J. Antimicrob. Chemother. 46, 11–18. doi: 10.1093/jac/46.1.11, PMID: 10882683

[ref46] Syed A. K. Ghosh S. Love N. G. Boles B. R. (2014). Triclosan promotes Staphylococcus aureus nasal colonization. MBio 5:e01015. doi: 10.1128/mBio.01015-13, PMID: 24713325 PMC3993860

[ref47] Wang X. Ouyang F. Feng L. Wang X. Liu Z. Zhang J. (2017). Maternal urinary Triclosan concentration in relation to maternal and neonatal thyroid hormone levels: A prospective study. Environ. Health Perspect. 125:067017. doi: 10.1289/EHP500, PMID: 28669941 PMC5743753

[ref48] Wen L. Duffy A. (2017). Factors influencing the gut microbiota, inflammation, and type 2 diabetes. J. Nutr. 147, 1468S–1475S. doi: 10.3945/jn.116.240754, PMID: 28615382 PMC5483960

[ref49] Wesgate R. Grasha P. Maillard J. Y. (2016). Use of a predictive protocol to measure the antimicrobial resistance risks associated with biocidal product usage. Am. J. Infect. Control 44, 458–464. doi: 10.1016/j.ajic.2015.11.009, PMID: 26810885

[ref50] Yang H. Wang W. Romano K. A. Gu M. Sanidad K. Z. Kim D.. (2018). A common antimicrobial additive increases colonic inflammation and colitis-associated colon tumorigenesis in mice. Sci. Transl. Med. 10:eaan4116. doi: 10.1126/scitranslmed.aan4116 29848663 PMC6343133

[ref51] Yee A. L. Gilbert J. A. (2016). MICROBIOME: is triclosan harming your microbiome? Science 353, 348–349. doi: 10.1126/science.aag2698, PMID: 27463658

[ref52] Yin Z. Liu Y. Yao Y. Li H. Qiao F. Wu J.. (2016). Influence of endogenous and exogenous estrogenic endocrine on intestinal microbiota in zebrafish. PLoS One 11:e0163895. doi: 10.1371/journal.pone.0167126, PMID: 27701432 PMC5049800

[ref53] Zhang Q. Wu Y. Wang J. Wu G. Long W. Xue Z.. (2016). Accelerated dysbiosis of gut microbiota during aggravation of DSS-induced colitis by a butyrate-producing bacterium. Sci. Rep. 6:27572. doi: 10.1038/srep27572 27264309 PMC4893749

[ref54] Zhang L. Xue X. Zhai R. Yang X. Li H. Zhao L.. (2019). Timing of calorie restriction in mice impacts host metabolic phenotype with correlative changes in gut microbiota. mSystems 4:e00348. doi: 10.1128/mSystems.00348-19 31796564 PMC6890928

[ref55] Zhou Z. Yang J. Chan K. M. (2017). Toxic effects of triclosan on a zebrafish (Danio rerio) liver cell line. ZFL. Aquat Toxicol. 191, 175–188. doi: 10.1016/j.aquatox.2017.08.009, PMID: 28843737

