# Supplementary material for: Effects of Long-Term Triclosan Exposure on Microbiota in Zebrafish
Source: Front Microbiol. 2021 Oct 12;12:604313. doi: 10.3389/fmicb.2021.604313 (PMC8546329; doi:10.3389/fmicb.2021.604313)
Supplement: Supplementary file 1 [file Data_Sheet_1.docx]

**Appendix 1:**

1.1 DNA extraction and PCR amplification

Microbial DNA was extracted from gastrointestinal tract samples using beads-beating method. 200mg sample was collected from frozen samples using CryoXtract CXT350 frozen sample aliquotter (Boston, MA). Each gastrointestinal tract sample was transferred to a 2mL screw-cap polypropylene microcentrifuge tube and the remaining material was frozen. sample was resuspended in 790μL lysis buffer (250μL 4M guanidine thiocyanate; 40μL 10% N-lauroyl sarcosine and 500μL 5% N-lauroyl sarcosine-0.1 M phosphate buffer [pH 8.0]). The 2mL tube was incubated at 70℃ for 1h. 750μL of 0.1mm-diameter silica beads was added, and the tube was shaken at 25Hz 10min in TissueLyser II (Qiagen). Polyvinylpolypyrrolidone (15mg) was added to the tube, which was vortexed and centrifuged for 5min at 14,000rpm. After recovery of the supernatant, the pellet was washed with TENP solution (50 mM Tris [pH 8], 20 mM EDTA [pH 8], 100 mM NaCl, 1% polyvinylpolypyrrolidone) and centrifuged for 5min at 14,000rpm. The new supernatant was added to the first supernatant. The washing step was repeated three times. The pooled supernatants (about 2mL) were centrifuged for 5min at 14,000rpm to remove particles and then split into two 2mL tubes. Nucleic acid was precipitated by the addition 1 volume of isopropanol for 10 min at 4℃ and centrifuged for 30min at 14,000rpm. Pellets were resuspended and pooled in 450μL of 100mM phosphate buffer (pH 8.0) and 50μL of 5M potassium acetate. The tube was placed on ice for 90 min and centrifuged at 14,000rpm for 30min. The supernatant was transfer to a new 1.5ml tube containing 3μL of Rnase A (10mg/mL) and incubated for 30min at 37℃. Nucleic acid was precipitated by the addition of 50μL of 3M sodium acetate and 1mL of absolute ethanol (pre-cooling at -20℃). The tube was incubated for 2h at -20℃. Nucleic acid was recovered centrifugation at 14,000rpm for 30min. The DNA pellets was finally washed by 70% ethanol pre-cooling at -20℃ (500μL three times), dried, and resuspended in 150μL of 10mM Tris-HCl (pH 8.0~8.5). The DNA concentration and purity were measured by Biodrop, and the size distribution of the DNA was estimated by electrophoresis.

The V3-V4 region of the bacteria 16S ribosomal RNA gene were amplified by PCR (94 °C for 3 min, followed by 21 cycles at 94 °C for 30 s, 55 °C for 30 s, and 72 °C for 30 s and a final extension at 72 °C for 8 min) using primers 341F 5’- CCTACGGGNGGCWGCAG-3’ and 805R 5’-GACTACHVGGGTATCTAATCC-3’. PCR reactions were performed in 25μL mixture containing 5 μL of 5 × FastPfu Buffer, 2 μL of 2.5 mM dNTPs, 1 μL of each primer (5μM), 0.5 μL of FastPfu Polymerase, and 10 ng of template DNA and PCR-grade water in a final volume of 20 μl.

Index PCR in order to distinguish different sample through different index sequence (Nextera XT Index Kit v2, illumina). Index PCR amplified by 95℃ 3min, followed by 8 cycles at 95℃ for 30s, 55℃ for 30s, and 72℃ for 30s and a final extension at 72℃ for 5min. PCR reactions were performed in 25μL mixture containing 5 μL of 5 × FastPfu Buffer, 2 μL of 2.5 mM dNTPs, 2.5 μL of index primers(4μM), 0.5 μL of FastPfu Polymerase, and 2.5μL of Ampolicons. Amplicons and index PCR production both were purified using agencourt AMPure XP Beads according to the manufacturer’s instructions.

1.2 Illumina MiSeq sequencing

After index PCR production purity, the production of purity was quantified using QuantiFluor -ST (Promega, U.S.). Purified amplicons were pooled in equimolar and paired-end sequenced (2 × 300) on an Illumina MiSeq platform (Illumina, San Diego, CA) according to the standard protocols.

**Appendix 2:**

Clean data was extracted from raw data based on the following criteria by using USEARCH 8.0: (i) Sequences of each sample were extracted using each index with zero mismatch. (ii) Sequences with overlap less than 50 bp were discarded. (iii) If the error rate of the overlap greater than 0.1, it was discarded. (iv) Sequences less than 400bp after merge were discarded. Quality-filtered sequences were clustered into unique sequences and sorted in order of decreasing abundance to identify representative sequences using Uparse according to Uparse OTU analysis pipeline, and singletons were omitted in this step. Operational Taxonomic Unit (OTUs) were classified based on 97% similarity after chimeric sequences removed using UPARSE (version 7.1 http://drive5.com/uparse/). The phylogenetic affiliation of each 16S rRNA gene sequence was analyzed by RDP Classifier (http://rdp.cme.msu.edu/) against the Silva (SSU123) 16S rRNA database using confidence threshold of 70%.

Sample diversity metrics were assessed on the basis of the nonparametic Shannon-Wiener (SW) diversity index and Simpson diversity index. The non-parametric Mann-Whitney U test was used to test for significant differences between two groups. Comparison of multiple groups was done using a nonparametric Kruskal-Wallis test. Bray-curtis, weighted and unweighted UniFrac were calculated in QIIME. The QIIME pipeline was also used to generate principal coordinate analysis (PCoA) plots based on the bray-curtis. PERMANOVA was used to test for statistical significance between the groups using 10,000 permutations (QIIME package). Bar plots, PCoA plots and receiver operating characteristics (ROC) curves and the area under the ROC curve (AUC) values were all generated in R (http://www.R-project.org/).
