# Supplementary material for: Effects of Long-Term Triclosan Exposure on Microbiota in Zebrafish
Source: Front Microbiol. 2021 Oct 12;12:604313. doi: 10.3389/fmicb.2021.604313 (PMC8546329; doi:10.3389/fmicb.2021.604313)
Supplement: Supplementary file 2 [file Data_Sheet_2.docx]

Manuscript title: Effects of Long-term TCS Exposure on Microbiota in Zebrafish

Authors: Ning Tang, MD^1#^, Pianpian Fan, MD^1#^, Xiaogang Yu, MD^1^, Rui Ma, MD^1^, Yexuan Tao, PhD^2^, Weiye Wang, PhD^1^, Fengxiu Ouyang, MD, MS, PhD^1*^

**^*^Correspondence:** Fengxiu Ouyang, Ministry of Education and Shanghai Key Laboratory of Children's Environmental Health, Xinhua Hospital, Shanghai Jiao Tong University School of Medicine, 1665 Kong Jiang Road, Shanghai 200092, China. Tel: +86-21-2507-8867, Email: [ouyangfengxiu@126.com](mailto:ouyangfengxiu@126.com); [ouyangfengxiu@xinhuamed.com.cn](mailto:ouyangfengxiu@xinhuamed.com.cn)

Number of pages: 9

Number of figures: 6

Number of tables: 2


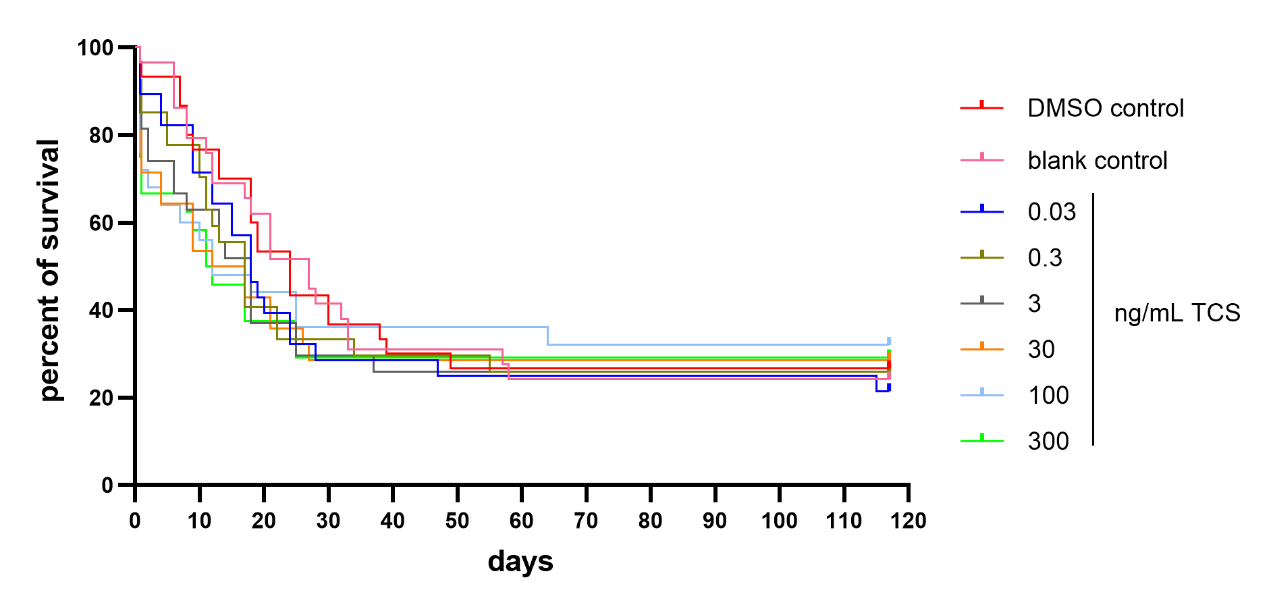


Figure S1. The Kaplan-Meier survival curves of zebrafish exposed to blank control, DMSO control and TCS concentrations from 0.03-300 ng/mL during 0-120 days.


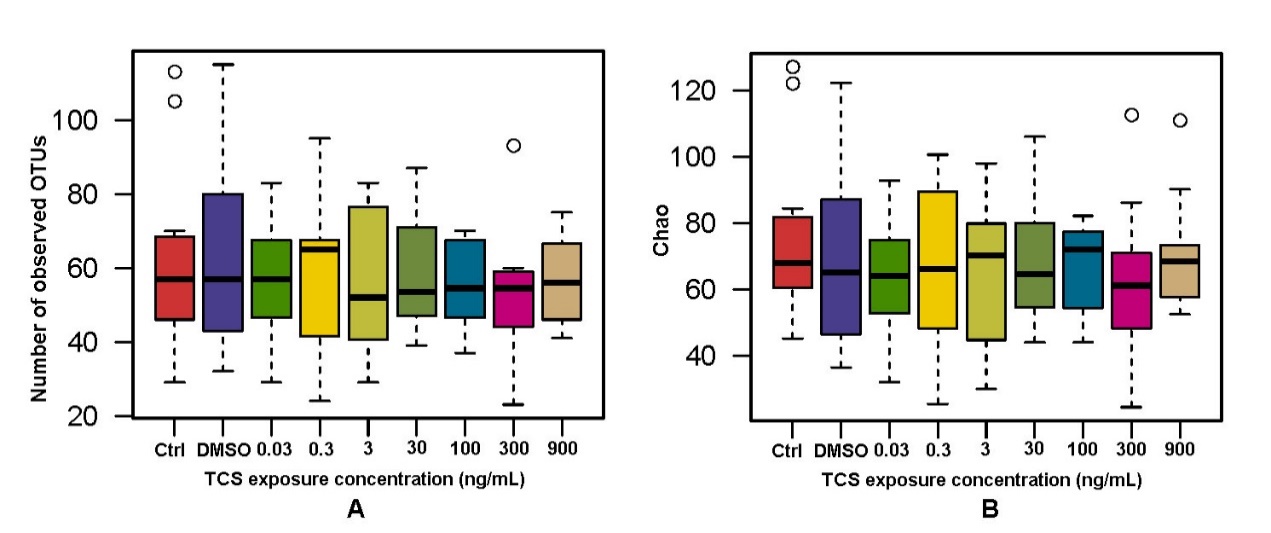


Figure S2. The alpha-diversity (A: number of observed OTUs; B: Chao index) of microbiota within gastrointestinal tract in adult zebrafish exposed to blank control, DMSO control and TCS concentrations from 0.03-900 ng/mL for 7 days.

Note: OTUs, Operational Taxonomic Units


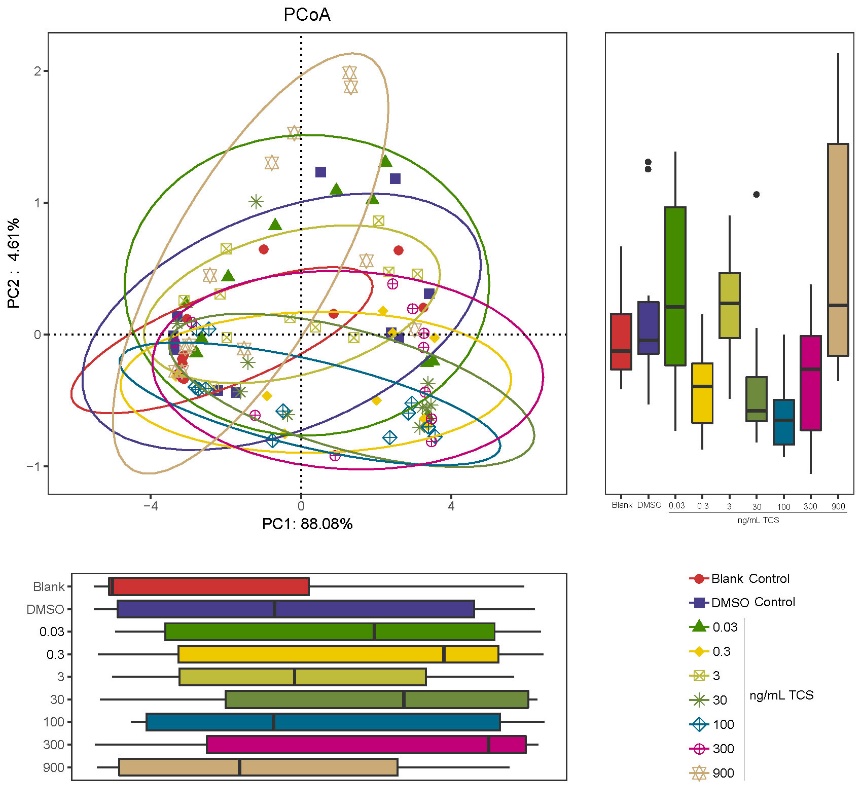


Figure S3. PCoA analysis of beta-diversity of microbiota within gastrointestinal tract in adult zebrafish exposed to blank control, DMSO control and TCS concentrations from 0.03-900 ng/mL for 7 days.


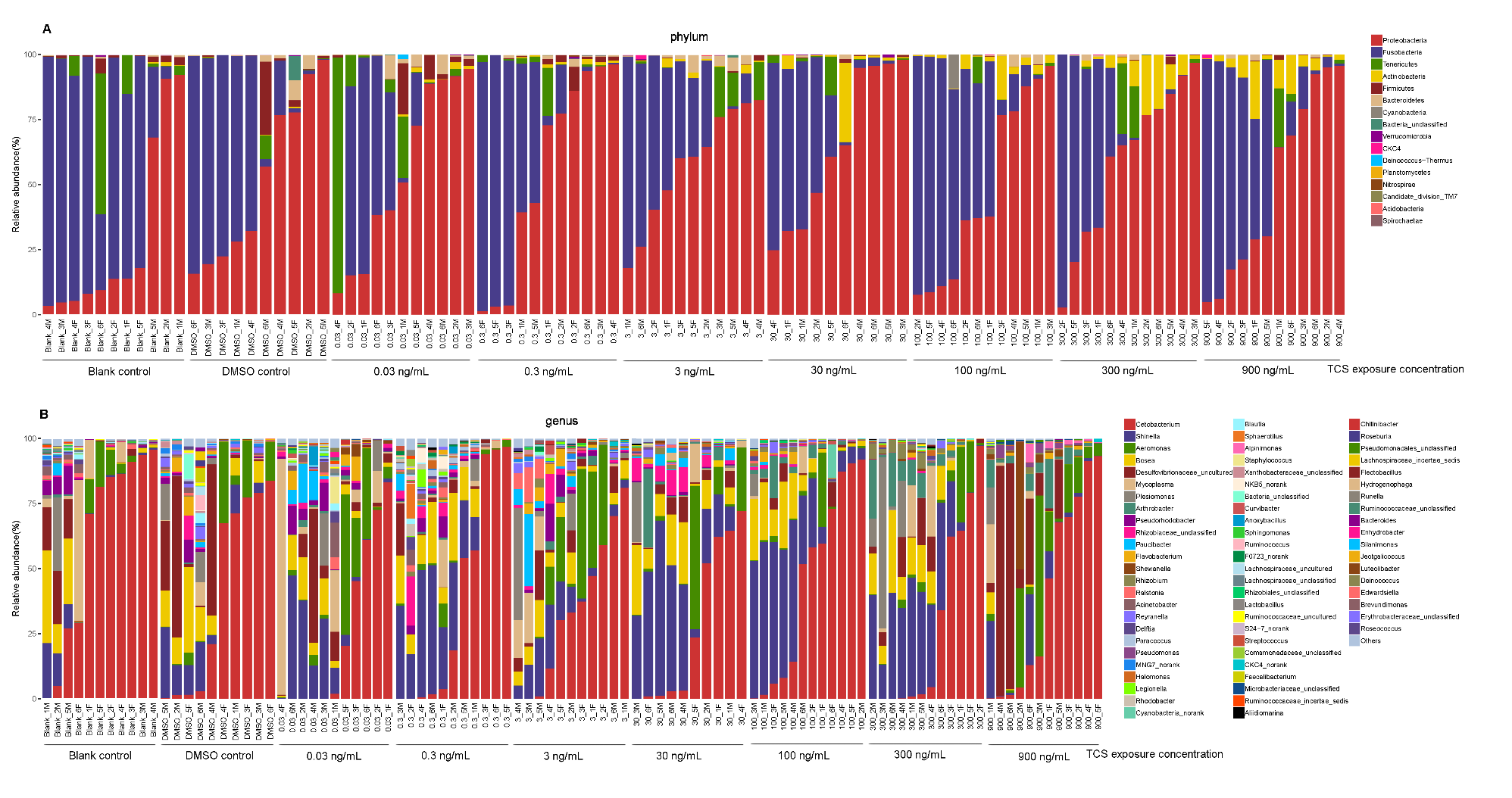


Figure S4. Composition of microbiota in gastrointestinal tract of adult zebrafish exposed to blank control, DMSO control and TCS concentrations from 0.03-900 ng/mL for 7 days at phylum (A) and genus (B) levels


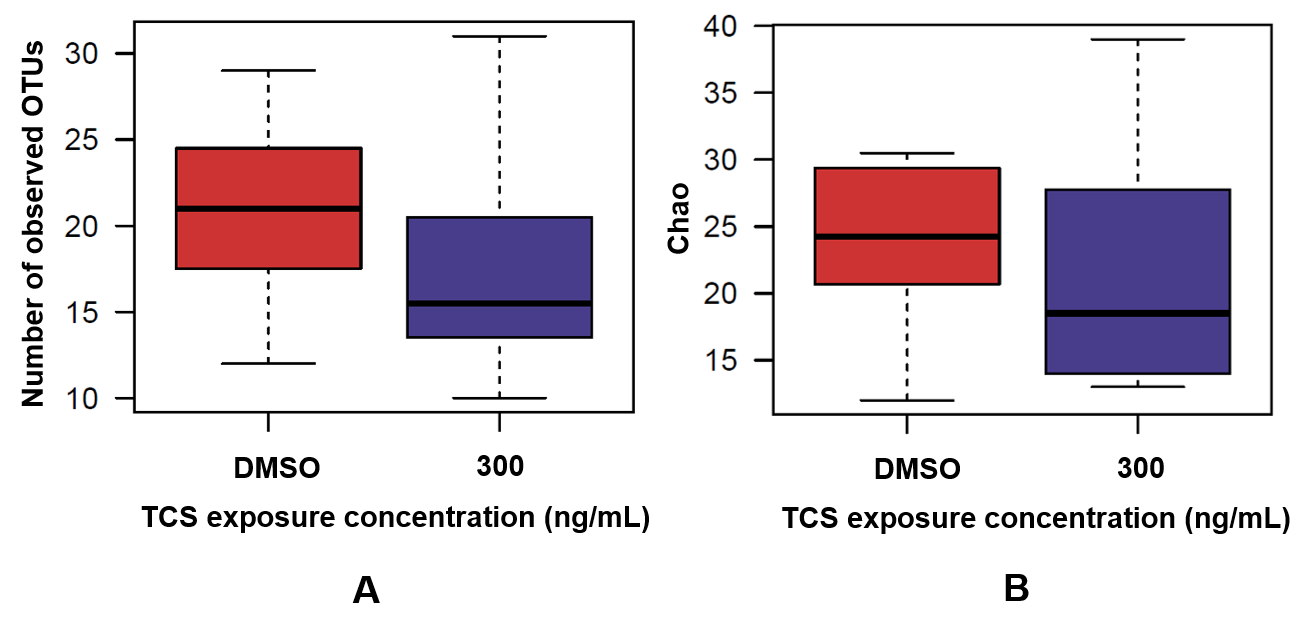


Figure S5. The alpha-diversity (A: number of observed OTUs; B: Chao index) of microbiota in zebrafish with long-term TCS 300 ng/mL exposure compared to DMSO group at 75 dpf

Note: OTUs, Operational Taxonomic Units


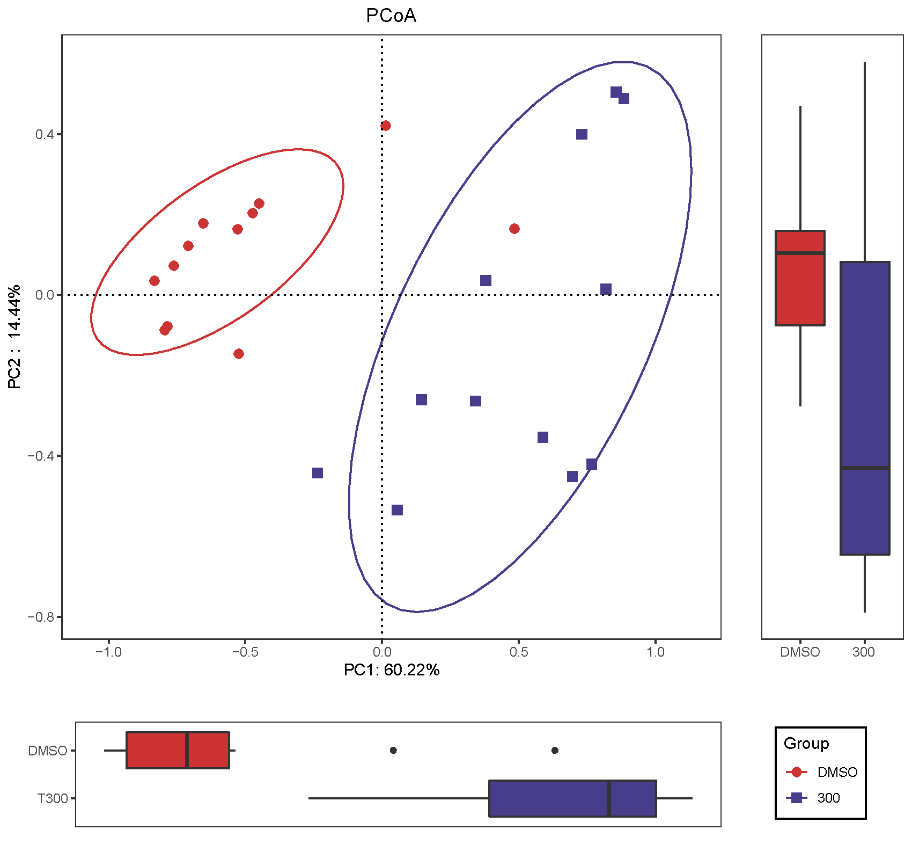


Figure S6. PCoA analysis of beta-diversity of microbiota in gastrointestinal tract in zebrafish at 75 dpf. Long-term TCS exposure at 300 ng/mL level was associated with separation on PC1 and PC2

|  |  | *Proteobacteria* | |  | *Fusobacteria* | |  | *Tenericutes* | |  | *Actinobacteria* | |
| --- | --- | --- | --- | --- | --- | --- | --- | --- | --- | --- | --- | --- |
| TCS exposure levels (ng/mL) | n | mean±SD | β (95%CI) |  | mean±SD | β (95%CI) |  | mean±SD | β (95%CI) |  | mean±SD | β (95%CI) |
| 0 (DMSO) | 10 | 0.5202±0.3208 | Reference |  | 0.4059±0.3775 | Reference |  | 0.0099±0.0283 | Reference |  | 0.0008±0.0018 | Reference |
| blank control | 11 | 0.2978±0.3540 | -0.2224 (-0.5086, 0.0639) |  | 0.6053±0.3737 | 0.1994 (-0.1134, 0.5122) |  | 0.0743±0.1618 | 0.0644 (-0.0372, 0.1660) |  | 0.0022±0.0040 | 0.0014 (-0.0415, 0.0443) |
| 0.03 | 11 | 0.5515±0.3384 | 0.0313 (-0.2550, 0.3175) |  | 0.2587±0.3321 | -0.1472 (-0.4600, 0.1656) |  | 0.1232±0.2699 | 0.1133 (0.0117, 0.2149)* |  | 0.0067±0.0083 | 0.0059 (-0.0370, 0.0487) |
| 0.3 | 11 | 0.5563±0.3899 | 0.0361 (-0.2502, 0.03223) |  | 0.3841±0.4208 | -0.0218 (-0.3346, 0.2910) |  | 0.0251±0.0536 | 0.0152 (-0.0864, 0.1168) |  | 0.0064±0.0064 | 0.0056 (-0.0373, 0.0484) |
| 3 | 11 | 0.5789±0.2234 | 0.0587 (-0.2276, 0.3449) |  | 0.3372±0.2877 | -0.0687 (-0.3815, 0.2441) |  | 0.0435±0.0737 | 0.0335 (-0.0681, 0.1351) |  | 0.0107±0.0134 | 0.0099 (-0.0330, 0.0528) |
| 30 | 10 | 0.6479±0.2982 | 0.1277 (-0.1654, -0.4207) |  | 0.2829±0.3080 | -0.1230 (-0.4432, 0.1971) |  | 0.0201±0.0460 | 0.0102 (-0.0938, 0.1141) |  | 0.0399±0.0940 | 0.0391 (-0.0048, 0.0830) |
| 100 | 12 | 0.4844±0.3503 | -0.0358 (-0.3163, 0.2447) |  | 0.4519±0.3697 | 0.0460 (-0.2605, 0.3526) |  | 0.0099±0.0295 | -0.0001 (-0.0996, 0.0995) |  | 0.0323±0.0455 | 0.0315 (-0.0106, 0.0735) |
| 300 | 12 | 0.5924±0.3023 | 0.0722 (-0.2083, 0.3527) |  | 0.2873±0.3701 | -0.1186 (-0.4252, 0.1879) |  | 0.0413±0.0916 | 0.0314 (-0.0682, 0.1309) |  | 0.0692±0.0780 | 0.0684 (0.0264, 0.1104)** |
| 900 | 12 | 0.5036±0.3567 | -0.0166 (-0.2971, 0.2639) |  | 0.4020±0.3785 | -0.0039 (-0.3104, 0.3027) |  | 0.0229±0.0645 | 0.0130 (-0.0866, 0.1125) |  | 0.0625±0.0672 | 0.0617 (0.0197, 0.1037)** |
| *P-*trend^§^ |  | 0.97 | |  | 0.87 | |  | 0.44 | |  | < 0.0001 | |
|  |  | *Firmicutes* | |  | *Bacteroidetes* | |  | *Cyanobacteria* | |  | *Bacteria_unclassified* | |
| TCS exposure levels (ng/mL) | n | mean±SD | β (95%CI) |  | mean±SD | β (95%CI) |  | mean±SD | β (95%CI) |  | mean±SD | β (95%CI) |
| 0 (DMSO) | 10 | 0.0336±0.0875 | Reference |  | 0.0187±0.0264 | Reference |  | 1.3×10^-6^±4.1×10^-6^ | Reference |  | 0.0095±0.0295 | Reference |
| blank control | 11 | 0.0150±0.0173 | -0.0186 (-0.0497, 0.0126) |  | 0.0031±0.0032 | -0.0156 (-0.0308, -0.0004)* |  | 1.2×10^-4^±3.0×10^-4^ | 0.0001 (-0.0112, 0.0114) |  | 0.0001±0.0002 | -0.0094 (-0.0175, -0.0013)* |
| 0.03 | 11 | 0.0343±0.0605 | 0.0007 (-0.0304, 0.0319) |  | 0.0231±0.0331 | 0.0044 (-0.0109, 0.0196) |  | 2.5×10^-4^±8.3×10^-4^ | 0.0003 (-0.0110, 0.0115) |  | 0.0001±0.0002 | -0.0094 (-0.0175, -0.0013)* |
| 0.3 | 11 | 0.0183±0.0263 | -0.0153 (-0.0464, 0.0158) |  | 0.0065±0.0095 | -0.0123 (-0.0275, 0.0030) |  | 1.2×10^-3^±2.6×10^-3^ | 0.0012 (-0.0101, 0.0125) |  | 0.00004±0.0001 | -0.0095 (-0.0176, -0.0014)* |
| 3 | 11 | 0.0031±0.0066 | -0.0305 (-0.0616, 0.0007) |  | 0.0223±0.0235 | 0.0036 (-0.0117, 0.0188) |  | 2.5×10^-6^±8.4×10^-6^ | 1.25×10^-6^ (-0.0113, 0.113) |  | 0.0009±0.0029 | -0.0086 (-0.0167, -0.0005)* |
| 30 | 10 | 0.0037±0.0042 | -0.0299 (-0.0618, 0.0019) |  | 0.0038±0.0057 | -0.0149 (-00305, 0.0007) |  | 0 | -1.3×10^-6^ (-0.0116, 0.0116) |  | 0.0001±0.0002 | -0.0095 (-0.0178, -0.0012)* |
| 100 | 12 | 0.0006±0.0007 | -0.0330 (-0.0635, -0.0025)* |  | 0.0093±0.0158 | -0.0094 (-0.0244, 0.0055) |  | 1.1×10^-2^±3.7×10^-2^ | 0.0110 (-0.00003, 0.0221) |  | 0.00004±0.0001 | -0.0095 (-0.0174, -0.0016)* |
| 300 | 12 | 0.0032±0.0086 | -0.0304 (-0.0609, 0.0001) |  | 0.0053±0.0077 | -0.0134 (-0.0284, 0.0015) |  | 0 | -1.3×10^-6^ (-0.0111, 0.0111) |  | 0.0003±0.0006 | -0.0092 (-0.0172, -0.0013)* |
| 900 | 12 | 0.0019±0.0049 | -0.0317 (-0.0622, -0.0011)* |  | 0.0054±0.0075 | -0.0133 (-0.0282, 0.0016) |  | 3.1×10^-5^±7.3×10^-5^ | 0.00003 (-0.0110, 0.0111) |  | 0.0001±0.0001 | -0.0095 (-0.0174, -0.0015)* |
| *P*-trend ^§^ |  | 0.004 | |  | 0.01 | |  | 0.60 | |  | 0.07 | |

Table S1. Effects of short-term 7-days TCS exposure on relative abundance of the top 8 dominant microbiota in the gastrointestinal tract of adult zebrafish at phylum level

Note: ^*^*P* < 0.05, ^**^*P* < 0.01, ^***^*P* < 0.0001; ^§^ *P*-trend for DMSO control and TCS concentrations from 0.03-900 ng/mL.

Table S2. Effects of short-term 7-days TCS exposure on relative abundance of the top 8 dominant microbiota in the gastrointestinal tract of adult zebrafish at genus level

|  |  | *Cetobacterium* | |  | *Shinella* | |  | *Aeromonas* | |  | *Bosea* | |
| --- | --- | --- | --- | --- | --- | --- | --- | --- | --- | --- | --- | --- |
| TCS exposure levels (ng/mL) | n | mean±SD | β (95%CI) |  | mean±SD | β (95%CI) |  | mean±SD | β (95%CI) |  | mean±SD | β (95%CI) |
| 0 (DMSO) | 10 | 0.4059±0.3775 | Reference |  | 0.0876±0.0910 | Reference |  | 0.0774±0.1113 | Reference |  | 0.0930±0.0972 | Reference |
| blank control | 11 | 0.6052±0.3736 | 0.1993 (-0.1135, 0.5121) |  | 0.0418±0.0714 | -0.0458 (-0.1852, 0.0937) |  | 0.0429±0.0631 | -0.0345 (-0.1523, 0.0833) |  | 0.0697±0.1226 | -0.0232 (-0.0969, 0.0505) |
| 0.03 | 11 | 0.2587±0.3321 | -0.1472 (-0.4600, 0.1656) |  | 0.1332±0.1714 | 0.0456 (-0.0938, 0.1851) |  | 0.1143±0.1806 | 0.0369 (-0.0809, 0.1547) |  | 0.0546±0.0605 | -0.0384 (-0.1121, 0.0353) |
| 0.3 | 11 | 0.3841±0.4208 | -0.0218 (-0.3346, 0.2910) |  | 0.2218±0.1839 | 0.1342 (-0.0053, 0.2736) |  | 0.0168±0.0260 | -0.0606 (-0.1784, 0.0573) |  | 0.1062±0.0915 | 0.0132 (-0.0605, 0.0869) |
| 3 | 11 | 0.3372±0.2877 | -0.0687 (-0.3815, 0.2441) |  | 0.0924±0.0852 | 0.0048 (-0.1346, 0.1443) |  | 0.1518±0.1901 | 0.0744 (-0.0434, 0.1923) |  | 0.0581±0.0428 | -0.0349 (-0.1086, 0.0388) |
| 30 | 10 | 0.2829±0.3080 | -0.123 (-0.4432, 0.1971) |  | 0.2864±0.2227 | 0.1988 (0.05604, 0.3415)** |  | 0.0946±0.1783 | 0.0172 (-0.1034, 0.1378) |  | 0.1068±0.0951 | 0.0139 (-0.0616, 0.0893) |
| 100 | 12 | 0.4519±0.3697 | 0.0460 (-0.2605, 0.3526) |  | 0.2898±0.2350 | 0.2022 (0.0655, 0.3388)** |  | 0.0320±0.0800 | -0.0454 (-0.1609, 0.0701) |  | 0.0971±0.0980 | 0.0042 (-0.0680, 0.0764) |
| 300 | 12 | 0.2873±0.3701 | -0.1186 (-0.4252, 0.1879) |  | 0.2594±0.1747 | 0.1718 (0.0352, 0.3085)* |  | 0.0535±0.0919 | -0.0239 (-0.1394, 0.0915) |  | 0.1011±0.0900 | 0.0081 (-0.0641, 0.0803) |
| 900 | 12 | 0.4020±0.3785 | -0.0038 (-0.3104, 0.3027) |  | 0.0607±0.1085 | -0.0269 (-0.1636, 0.1097) |  | 0.1373±0.1864 | 0.0599 (-0.0555, 0.1754) |  | 0.0081±0.0233 | -0.0849 (-0.1571, -0.0127)* |
| *P*-trend^§^ |  | 0.88 | |  | 0.24 | |  | 0.85 | |  | 0.27 | |
|  |  | *Desulfovibrionaceae_uncultured* | |  | *Mycoplasma* | |  | *Plesiomonas* | |  | *Arthrobacter* | |
| TCS exposure levels (ng/mL) | n | mean±SD | β (95%CI) |  | mean±SD | β (95%CI) |  | mean±SD | β (95%CI) |  | mean±SD | β (95%CI) |
| 0 (DMSO) | 10 | 0.1506±0.2508 | Reference |  | 0.0099±0.0283 | Reference |  | 0.0293±0.0531 | Reference |  | 0.0006±0.0016 | Reference |
| blank control | 11 | 0.0504±0.0804 | -0.1002 (-0.2344, 0.0340) |  | 0.0743±0.1618 | 0.0644 (-0.0372, 0.1660) |  | 0.0270±0.0845 | -0.0024 (-0.0836, 0.0789) |  | 0.0018±0.0037 | 0.0012 (-0.0400, 0.0424) |
| 0.03 | 11 | 0.0793±0.1487 | -0.0713 (-0.2055, 0.0629) |  | 0.1232±0.2699 | 0.1133 (0.0117, 0.2149)* |  | 0.0298±0.0666 | 0.0005 (-0.0807, 0.0817) |  | 0.0058±0.0086 | 0.0052 (-0.0360, 0.0464) |
| 0.3 | 11 | 0.0257±0.0600 | -0.1248 (-0.2591, 0.0094) |  | 0.0251±0.0536 | 0.0152 (-0.0864, 0.1168) |  | 0.0075±0.0248 | -0.0218 (-0.1030, 0.0594) |  | 0.0037±0.0033 | 0.0031 (-0.0382, 0.0443) |
| 3 | 11 | 0.0454±0.0596 | -0.1052 (-0.2394, 0.0290) |  | 0.0435±0.0737 | 0.0335 (-0.0681, 0.1351) |  | 0.0571±0.1308 | 0.0278 (-0.0535, 0.1090) |  | 0.0085±0.0133 | 0.0080 (-0.0333, 0.0491) |
| 30 | 10 | 0.0320±0.0343 | -0.1186 (-0.2559, 0.0188) |  | 0.0201±0.0460 | 0.0102 (-0.0938, 0.1141) |  | 0.0237±0.0721 | -0.0056 (-0.0887, 0.0776) |  | 0.0380±0.0943 | 0.0374 (-0.0048, 0.0796) |
| 100 | 12 | 0.0208±0.0323 | -0.1298 (-0.2613, 0.0017) |  | 0.0099±0.0295 | -0.0001 (-0.0996, 0.0995) |  | 0.0071±0.0243 | -0.0222 (-0.1018, 0.0574) |  | 0.0295±0.0445 | 0.0290 (-0.0114, 0.0694) |
| 300 | 12 | 0.0471±0.0680 | -0.1034 (-0.2350, 0.0281) |  | 0.0413±0.0916 | 0.0314 (-0.0682, 0.1309) |  | 0.0839±0.1922 | 0.0546 (-0.0250, 0.1342) |  | 0.0670±0.0780 | 0.0665 (0.0261, 0.1068)** |
| 900 | 12 | 0.2101±0.3275 | 0.0595 (-0.0720, 0.1910) |  | 0.0229±0.0645 | 0.0130 (-0.0866, 0.1125) |  | 0.0121±0.0410 | -0.0173 (-0.0968, 0.0623) |  | 0.0460±0.0554 | 0.0455 (0.0051, 0.0858)* |
| *P*-trend^§^ |  | 0.78 | |  | 0.44 | |  | 0.76 | |  | 0.0003 | |

Note: ^*^*P* < 0.05, ^**^*P* < 0.01, ^***^*P* < 0.0001; ^§^ *P*-trend for DMSO control and TCS concentrations from 0.03-900 ng/mL.
